# Characterization of Laser Cleaning of Artworks

**DOI:** 10.3390/s8106507

**Published:** 2008-10-23

**Authors:** Jan Marczak, Andrzej Koss, Piotr Targowski, Michalina Góra, Marek Strzelec, Antoni Sarzyński, Wojciech Skrzeczanowski, Roman Ostrowski, Antoni Rycyk

**Affiliations:** 1 Institute of Optoelectronics, Military University of Technology, 2 Gen. S. Kaliskiego Str., 00-908 Warsaw, Poland; E-Mails: jmarczak@wat.edu.pl (J. M.); asarzynski@wat.edu.pl (A. S.); wskrzeczanowski@wat.edu.pl (W. S.); rostrowski@wat.edu.pl (R. O.); 2 Academy of Fine Arts in Warsaw, Wybrzeze Kościuszkowskie 37, 00-379 Warsaw, Poland; E-Mail: kossa@asp.waw.pl (A. K.); 3 Institute of Physics, Nicolaus Copernicus University, ul. Grudziądzka 5, 87-100 Toruń, Poland; E-Mail: ptarg@phys.uni.torun.pl (P. T.)

**Keywords:** Laser cleaning, laser spectroscopy, artwork diagnostics

## Abstract

The main tasks of conservators of artworks and monuments are the estimation and analysis of damages (present condition), object conservation (cleaning process), and the protection of an object against further degradation. One of the physical methods that is becoming more and more popular for dirt removal is the laser cleaning method. This method is non-contact, selective, local, controlled, self-limiting, gives immediate feedback and preserves even the gentlest of relief - the trace of a paintbrush. Paper presents application of different, selected physical sensing methods to characterize condition of works of art as well as laser cleaning process itself. It includes, tested in our laboratories, optical surface measurements (e.g. colorimetry, scatterometry, interferometry), infrared thermography, optical coherent tomography and acoustic measurements for “on-line” evaluation of cleaning progress. Results of laser spectrometry analyses (LIBS, Raman) will illustrate identification and dating of objects superficial layers.

## 1. Introduction

Commonly used methods of surface cleaning in conservation of art works are based on mechanical or chemical techniques which are individually selected by experienced conservators. These traditional methods are very difficult to control. Cleaning of delicate objects, diverse from the point of view of materials composition needs not only extended expert appraisements of used substances, but also minimization of possible damages, always present in the case of mechanical cleaning. Chemical reagents show similar interactions in the conservation of paintings, where chemicals penetrate technological painting layers and causes permanent, difficult to analyze, cross-sectional alterations. Conservation practice shows the necessity of frequent treatments of sophisticated objects with complex technological structures and individual preservation states, resulting from the influence of diverse external factors, as well as changes in original material on construction itself. Application of conventional conservation methods is limited and difficult. Moreover, every detail requires individual, predetermined cleaning parameters. Application of laser technique gives possibility of almost full control of the encrustation removal process at the surface of art works. Selective and precise interaction of the light beam is a fundamental advantage of non-invasive treatment of more or less tightly connected unwanted surface layers. Specific properties of lasers, decrease of system costs, and reduction of dimensions of laser cleaning systems have contributed to increasing applications of lasers in conservation, particularly during recent the ten to fifteen years [1-6]. Laser cleaning must be considered as an advanced tool applied in cases where traditional techniques may be inadequate. Nevertheless, extreme care should be taken for the optimization of the operational parameters in order to ensure the absence of any negative effects induced on the artwork.

It should be pointed out in this place that laser cleaning of historical objects is still far from being as popular as the conventional techniques, being employed in an increasing, but relatively small number of restoration interventions, mainly applied to stone substrates. Even if the last generation of laser systems has improved the understanding of their effects and their engineering, laser cleaning is not yet a mature technology for earlier restoration tests, there is also lack of in-depth knowledge of the basic laser-artwork interaction mechanisms. There is still also a lack of diagnostic devices providing qualitative and quantitative information during the laser cleaning intervention.

Application of laser radiation in physico-chemical surface analyses and structural objects investigations, have started simultaneously with development of laser cleaning systems. In the face of increasing interest in laser cleaning and diagnostic systems, important is acquaintance of the conservation community with the fundamental advantages and shortcomings of laser radiation in treatment and analysis of matter. Material investigations have shown that main conservation cleaning problem with stone sculptures exposed to atmospheric pollution is the preservation of delicate patinas. This historical superficial layer can be lost, as a result, to the use of non-laser, aggressive, not fully controlled cleaning methods.

In the present paper, authors describe and discuss selected areas of applications of lasers and optoelectronics in conservation of monuments and works of art, with particular attention paid to noninvasive, physico-chemical and structural analytical methods. Different kinds of sensors play substantial roles in the presented measurement systems, especially in detection of diffusively reflected, sampling or emitting radiation. The data presented are based on fifteen years of experience in laser cleaning and diagnostics of dozens priceless objects in Poland, France and Croatia [7-9].

## 2. Influence of environment

Increasing pollution levels of monuments and sculptures exhibited in the open air inside built-up areas are the result of environmental pollution, composed mainly of soot and dust emitted by industrial objects or rising into the air from the earth surface. The next pollution groups originate from motor exhaust gases and substances generated by modern industry. Their influence on the environment, including biological effects, is by far stronger than in the case of particulates. Effective detection, counteraction and removal are respectively much more difficult, in some cases unknown are efficacious restoration procedures. Over one hundred polluting substances identified so far can be grouped into the following categories:

- hydrocarbons, emitted during non-complete combustion of oil-derivative products,
- carbon monoxide, constituting around 2/3 of all volatile poisons originating from motor exhaust fumes,
- nitrogen and sulphur oxides (SO_2_ - dozens of millions tons per year in Europe).

Well known consequences of all those processes are aggressive acid rains, polluting environment and destructing material and cultural objects. A good example is surface arising of calcium sulphate (from calcium carbonate and sulphuric acid) which forms impermanent plaster, and carbonic acid, decomposing later into water and carbon dioxide.

**Figure 1.**
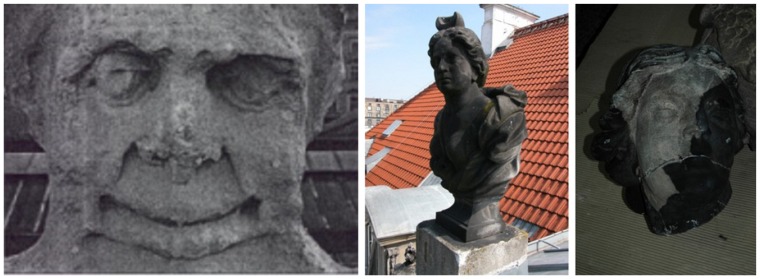
Illustration of destructive influence of acid rains on sandstone sculptures: a) Drapper's Hall in Cracow (1995); b) bust at Czapski Palace, Warsaw, seat of Academy of Fine Arts; c) woman's head during laser cleaning.

The present fast degradation of human natural environment has ocurred over 200 years, but the main changes were the result of the last seventy years or so of neglected control of industrial development. The percentages of artwork surface associated with corrosion damage (Figure 1) reaches yearly 3 to 5 % in dependence on monument localization and atmosphere pollution [10,11].

The phenomenon of stone destruction grows with an accelerated rate as a result of larger atmosphere pollution. Marble, sandstone or limestone sculptures deteriorate in many urbanized landscapes all over the world, as a rule in the following order:

- darkening of stone surface and conversion into calcium sulphate,
- swelling of top layers,
- shattering of superficial layers and further, deeper degradation,
- dump absorption (acid rains) in uncovered, fresh subsequent layer.

Beside the artistic and aesthetic depletion of the artwork, deposits and encrustation can cause further degradation processes of both a physical and a chemical nature, requiring prompt restoration interventions before the artistic content of the stonework is irremediably lost [12]. Such an active conservation include surface cleaning, which is often one of the first actions to be undertaken. It represents a crucial step in the whole restoration procedure, as the effects of this operation are irreversible and influence the future conservation of the restored artworks. Cleaning itself is also an important part of the artwork stabilizing process and is one of the most important processes in the active conservation of artifacts, preparing possible further treatments if needed: consolidation, coating of a surface or reconstruction of totally damaged elements.

The cleaning technique(s) employed should provide a high selectivity in order to discriminate the original substrate from the degraded layers, and to preserve the patina when present. Removal of different materials from an artifact is very difficult to control and the results can be highly critical for the long term preservation of the item. Careless cleaning can worsen appearance of work of art or cause its further damage, which can lead to the accelerated destruction process.

Prevention is always better than the treatment. Fundamental knowledge concerning influence of environment on preservation of art works is necessary to prevent deterioration and degradation of any historical object. Environment is diverse, with continuously variable pollution dynamics, supplementary and additive different effects. Table 1 shows main atmosphere polluting substances and summarizes their influence on the preservation of the cultural heritage objects.

Atmospheric pollution influences art works in different way and with a rate dependent on changes in environmental humidity and temperature. Interaction of air contaminants with historical objects requires a wide range of investigations. So far, much research has been directed at understanding global and long term effects. However, it has been proven that some pollutants, especially NO_x_, SO_2_ and some hydrocarbons, are the main air components responsible for degradation of monuments and works of art. Threshold values of these substances recommended for museums and galleries are much lower than those accepted as harmless for human health for the open air. Concentrations of atmospheric pollutants should be therefore under control, through periodic measurements or continuous monitoring. Measurement of air gaseous contaminants at the level of parts per billion (ppb) is a difficult and complex task. It can be realized by collection of samples and laboratory analyses, using sophisticated physico-chemical techniques inaccessible *in situ*. Good examples are conventional gas tubes, collecting air samples over specified periods of time, and periodically tested in the laboratory. The preference given to such averaging tests results from the lack of real-time cheap, reliable measurement techniques. One of the solutions could be laser and optoelectronic techniques utilizing various processes of light-matter interaction, including e.g. absorption of laser light, fluorescence or Raman backscattering. However, methods based on laser spectroscopy seem to be too complex and expensive. At the other side, progress in construction of miniature tunable laser diodes or LEDs promises fast implementation of compact, cheap and sensitive absorption sensors, based on DIAL concept (differential absorption lidar).

## 3. Diagnostics of art works

### 3.1 Non-destructive, physico-chemical and structural methods of analysis of monuments and works of art

The main aim of analysis of monuments and works of art is identification of artwork structure, its important chemical components as well as characterization of its preservation state, including determination of the influence of external factors. It is well known, that the nature of a given conservation problem prescribes future utilization of specified technology or a combination of several techniques. Therefore, many powerful analytical methods developed as a result of technological progress in optoelectronics are addressed now to solve different and complex problems, arising in art conservation. It applies to different conservation studies, from identification of substrate and top layer materials (pigments, painting media, varnishes), through mapping of structural defects and mechanical discontinuities, up to dating, authenticity studies and careful removal of unwanted layers (encrustation, overpainting, old varnish etc.). Obviously, the most safe for objects are noninvasive techniques *in situ* (if possible to apply).

**Figure 2.**
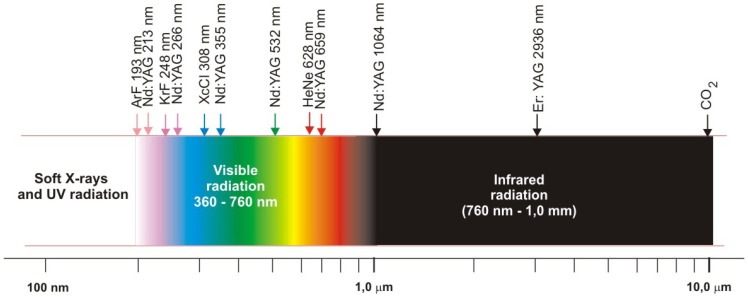
Most commonly lasers used in conservation of artworks. Location of laser wavelengths inside electromagnetic radiation spectrum.

After about fifty years of the laser R&D, more than 10,000 laser transitions are known. Figure 2 shows electromagnetic radiation spectrum in the range of 200 – 10 000 nm, with marked place of laser wavelengths, most commonly used in conservation.

Particularly popular analytical methods have become spectroscopic techniques (laser and non-coherent), mainly due to their sensitivity, flexibility and analytical methodology [14-16]. Spectroscopy delivers information, which is directly or indirectly connected with chemical nature of investigated materials. Wide application in the diagnostics of historical object found classical Fourier infrared spectrometry (FTIR) or its DRIFT variety with the utilization of diffuse reflection of radiation. Range of FTIR spectroscopy applications include:

identification of molecular compounds created at the artwork surface,
studies of composition of painting layers,
identification of fibers material, chemical composition and soiling of paper and parchment,
investigations of epoxy resins.

Optical measurement methods (scaterrometry, shadowgraphy, microscopy, reflectometry) are commonly supplementing sets of diagnostic methods. Increases interest in application of multispectral imaging for evaluation the results of laser cleaning, identification and mapping of painting materials and visualization of top surface layers.

Diagnostic techniques that utilize X-ray radiation and methods of nuclear physics and chemistry are also supporting conservation of artworks. The most popular is scanning electron microscopy (SEM), frequently with radiation energy dispersion (EDR or EDX). Chemical and crystallographic surface modifications, composition and volume structure of pigments and other materials are studied with the use of X-ray diffraction and fluorescence. Additional basic materials research is sometimes realized using complex systems of mass spectrometry and atomic force microscopy.

**Figure 4.**
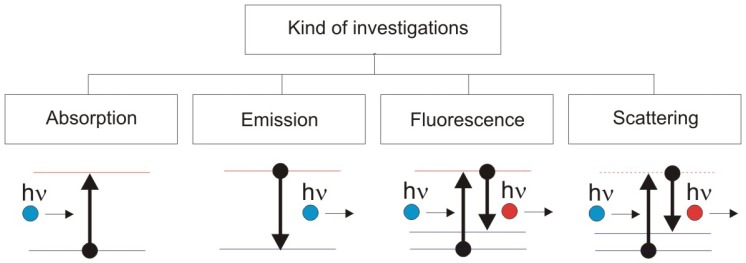
Measurement methods based on characteristic photon interaction and spectra of objects.

Practically all laser and optoelectronic methods involved in physico-chemical and structural studies of artworks are summarized in Table 2. Table 2 illustrates natural transfer of different laser devices to the domain of artwork diagnostics, especially after development of modern, compact, portable and reliable laser sources. Figure 4 illustrates schematically measurements of chemical composition of historical objects, based on absorption, emission, fluorescence and scattering of laser radiation during its interaction with matter.

The most important advantage of laser methods presented in Table 2 is their non-destructiveness or micro-destructiveness (LIBS). Laser measurements can be performed both *in-situ* and, in many cases *on-line*.

### 3.2 Laser methods

#### 3.2.1. Laser induced breakdown spectroscopy (LIBS) and Raman spectroscopy

Laser induced breakdown spectroscopy LIBS is utilized for determination of elemental composition and stratigraphy of different art works: easel and mural paintings, sculptures, building materials, minerals as well as different objects with multilayer structure.

In short, the LIBS method consists of laser evaporation of a minimal amount of the investigated material in the form of a plasma cloud emitting continuous and linear optical radiation. Analysis of linear plasma radiation allows determination of elements in the sample. LIBS can be also used in the automation of laser cleaning process. The procedure is based on registration of two preselected lines: one – characteristic for removable layer, and second – usually characteristic for the original object substrate. Decrease in the ratio of those line intensities determines the level of cleaning. Crossing of predetermined threshold value stops laser, moves beam to the adjacent area and reactivates cleaning process.

**Figure 5.**
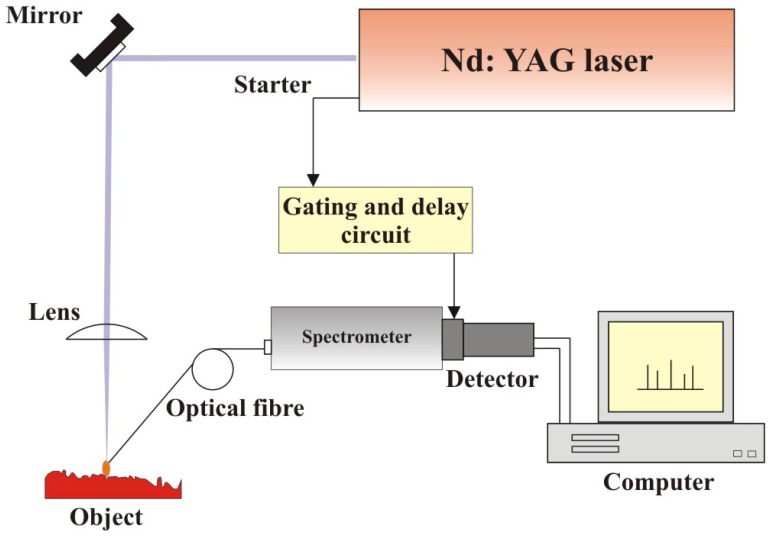
Block scheme of LIBS experimental setup.

Figure 5 shows experimental setup used in all investigations presented in the paper. It consists of a Mechelle 900 spectrometer with a SensiCamFS camera, characterized by spectral resolution λ*/*Δλ = 900 and spectral measurement range 300 - 1000 nm. Period of LIBS spectra registration was usually in the range of 0.5 to 10 μs with time delay 300 – 1000 ns after the beginning of laser sampling pulse (domination of linear radiation in total spectrum). Pulse Nd:YAG laser (Quantel, model BRIO) with fundamental wavelength 1064 nm has been utilized as an excitation source.

**Figure 6.**
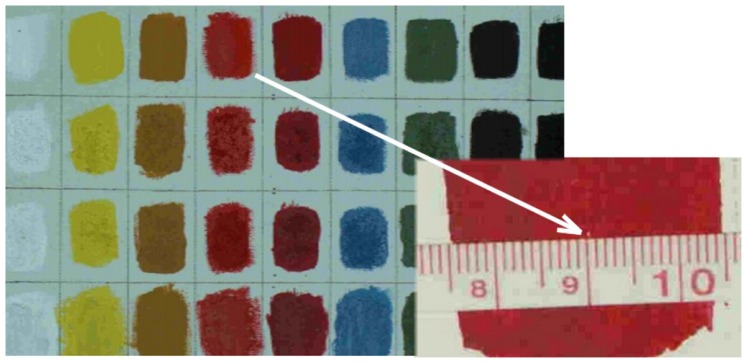
White arrow shows photograph of a crater produced by 20 laser pulses (pulse energy 30 mJ) in acryl painting layer with cadmium red pigment.

In principle, due to micro-sampling character of measurement, LIBS can be treated as an invasive technique, causing micro-damages of objects. However, careful action can produce very small crater, even invisible with the naked eye. Figure 6 shows the photographic image of a shallow crater after 20 laser pulses of 30 mJ. Its diameter is below 0.3 mm, important is also its minimal depth. Unavoidable damages could be really small and difficult to detect.

**Figure 7.**
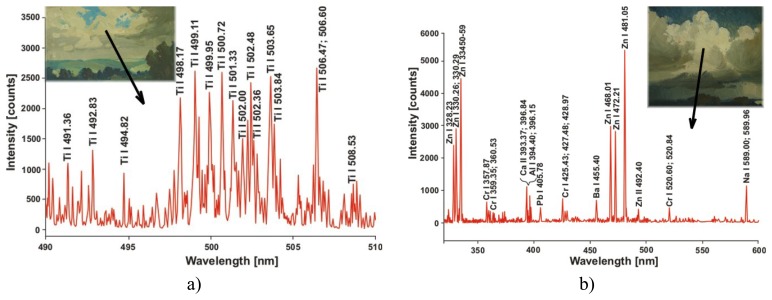
Stanisław Żukowski landscape paintings - identification of white pigments: a) titanium white; b) zinc white.

The identifying pigment LIBS measurements can be also used for painting dating, in spite of the fact that such dating is not always unambiguous. Among others, several Stanisław Żukowski landscapes (from a set of unnamed artworks) were investigated. The resulting LIBS spectra of two white fragments (clouds) are shown in Figure 7.

Titanium white pigment (Figure7a) was developed in 1920, which almost exactly establishes the time of painting at the turn of the 19^th^ and 20^th^ centuries. At the other hand, zinc white pigment was used from 1830, which leaves ambiguity in the time domain. However, the similar theme and artist's expression suggest a time localization of both paintings after 1920.

**Figure 8.**
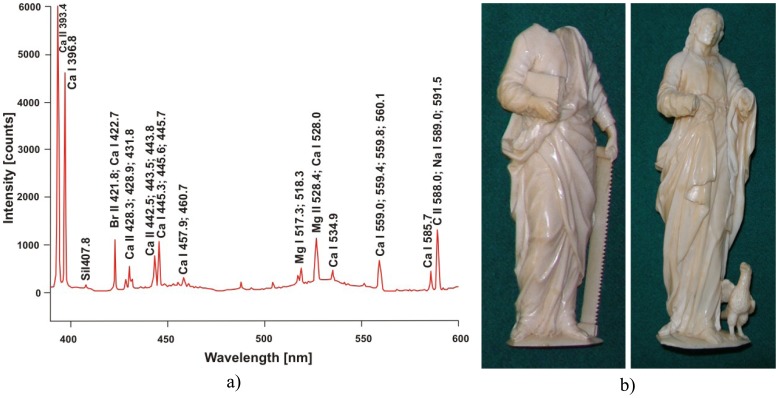
a) Ivory LIBS spectrum. b) Photographs of figures of St.Simon – left, and St.John – right side.

**Figure 9.**
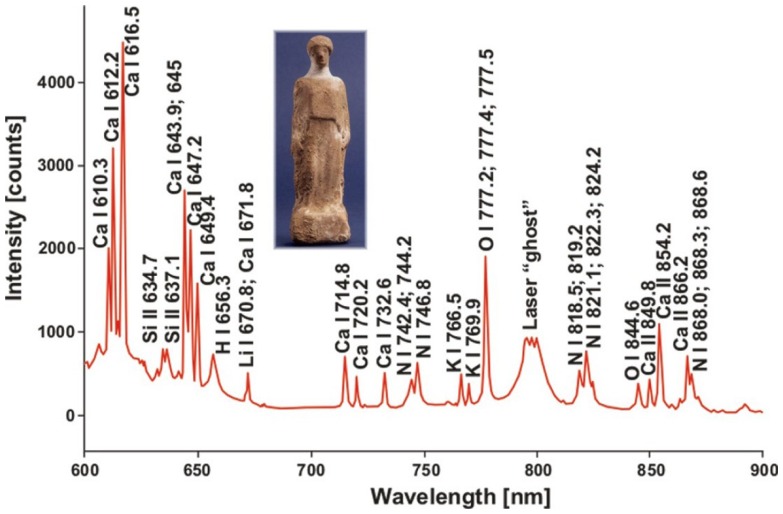
LIBS spectrum of ancient terracotta figure (shown at small photograph).

LIBS spectroscopy has been utilized also during studies of two ivory sculptures of apostles – St. Simon and St. John (property of Wawel Treasure in Cracow) and a human figure, probably made of terracotta from the beginning of Christian era (property of Archaeological Museum in Poznań). In case of the ivory figures (Figure 8), LIBS spectra are almost identical, with small difference only in the elementary peaks intensity. However, this information is inadequate to state that they have been carved using the same ivory piece. Identification of elemental composition of ancient sculpture material presented in Figure 9 allowed conservators to select appropriate and safe conservation procedure.

**Figure 10.**
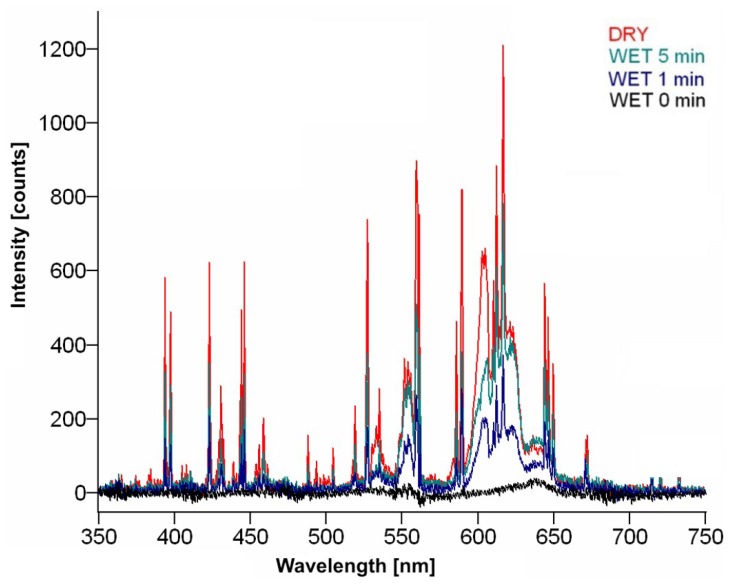
Comparison of emission spectra for sandstone encrustation during dry and wet laser cleaning.

Laser cleaning process can be realized in dry or wet regime. During wet laser cleaning, the removed layer is sprayed with water or other liquid. Theoretical calculations as well as experiments [19] have confirmed the increase of cleaning rate in case of a wet object surface. Equivalent LIBS spectra for a sandstone sample are shown in Figure 10. Significant decrease of spectral lines intensity can be seen after water spraying of encrustation, which reflects the reduction of plasma temperature. It can be explained through deeper penetration of laser radiation into the wet encrustation layer. The same laser energy is heating much larger volume of material, which causes the temperature decrease. Passage of time causes water evaporation and subsequent increase of peaks intensities.

**Figure 11.**
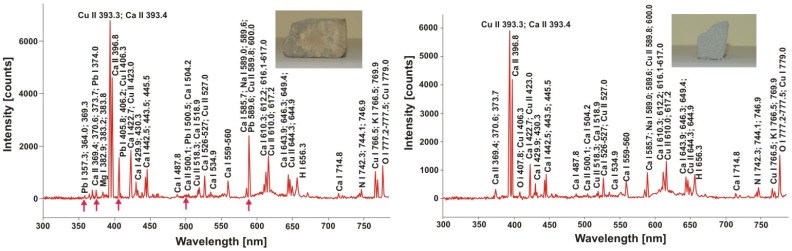
LIBS spectra of Myślenicki sandstone from Sigismund Chapel, Cracow. a) encrustation with lead lines (red arrows); b) “fresh” sandstone sample.

Laser technology has been included into many conservation programs, also into the program of conservation and restoration of internal décor of Sigismund Chapel at Wawel Hill in Cracow, about 800 m^2^ of decorative sculptor's surfaces made of green-gray Myślenicki sandstone at the beginning of the 16th century. Figure 11 shows LIBS spectra of original sandstone sample from the Sigismund Chapel and the same sample after breaking. Characteristic for “civilization” are lead lines, observed in the spectrum of polluted sandstone and absent in the “fresh” surface spectrum.

**Figure 12.**
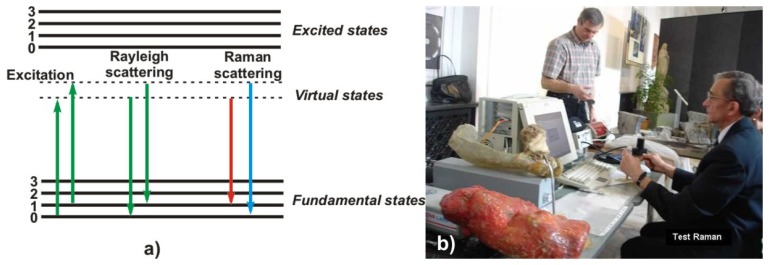
a) Schematic illustration of energy levels involved in Raman phenomenon. b) Laboratory Raman stand during tests of modern sculptures.

**Figure 13.**
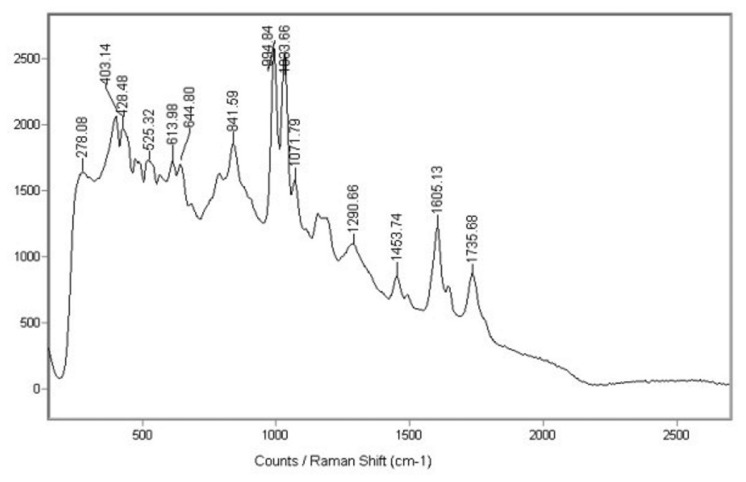
Characteristic Raman spectrum of polyester (AKEMI Marmokitt 1000) -substrate of sculpture “Journey” of Alina Szapocznikow.

Raman spectroscopy is a light scattering technique, and can be thought of in its simplest form as a process where a photon of light interacts with a sample to produce scattered radiation of different wavelengths (Figure 12a). Molecular Raman spectroscopy is particularly well-suited technique for the determination of the artistic materials in a non-destructive way. By focusing a low power laser on a sample the intensity of the inelastically scattered light is plotted against the Raman wavenumber, which is proportional to the difference in energy between the laser and the scattered light. Figure 12b shows photograph of experimental stand during identification of substrate materials in modern sculptures of Alina Szapocznikow. An example result is shown in Figure 13 – identification of polyester (AKEMI Marmokitt 1000 Transparent Wasserhell polyester) as a building material in the sculpture “Journey”.

#### 3.2.2. Optical Coherent Tomography (OCT)

The OCT method allows noncontact and nondestructive imaging of semitransparent objects, mainly applied to the analyses of paintings restoration. It enables fast and convenient calibration of ablation conditions for the particular laser-varnish combination [20]. Moreover, the technique provides information on the volume of removed material and the thickness, structure, and quality of the remaining varnish layer [21]. This is essential to control the ablation depth.

**Figure 14.**
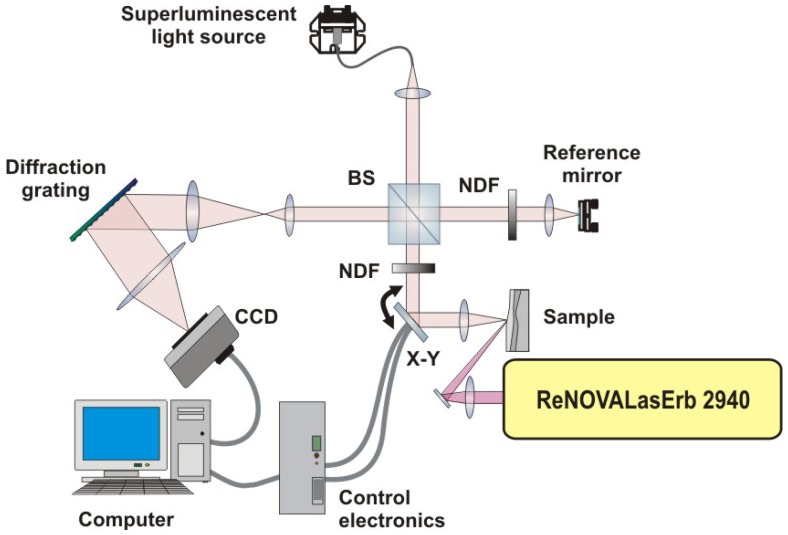
Diagram of the SOCT instrument used in the study: BS—beam splitter, NDF— neutral density filter, CCD – CCD linescan camera, X-Y – galvanometric scanner.

The spectral OCT (SOCT) system shown schematically in Figure 14 is a bulk optics instrument based on a Michelson interferometer setup. The superluminescent diode (Superlum Ltd., Russia) emitting light with a central wavelength of 835 nm and spectral width (FWHM) of 50 nm was used as a light source. The beam of light is split into two interferometric arms with the aid of a 50:50 nonpolarising beam-splitting cube BS. One arm is terminated by the reference mirror kept in a fixed position. The other, namely, the object arm, comprises a set of two transversal scanners *X*-*Y* (Cambridge Technology Inc., USA) which are used for scanning the probing beam across the object. The beam is focused on the object by a lens L and penetrates the object. Some of it is scattered and/or reflected back from elements in its structure, and this is finally collected by the same optics L, and returned to the beam-splitter BS. It is then combined with the light returning from the reference arm. The resulting interference signal is analyzed and registered by a spectrometer (Spectrogon AB, Sweden). The spectral fringe patterns registered by this detector are then transferred to a personal computer. The fringe pattern signal is then reverse Fourier transformed into one line of a tomogram (an A-scan). The exposure time per A-scan is usually 30 microseconds. The axial resolution of the system is around 6 μm in these media which have refractive indices ranging from 1.3 to 1.5. The transversal resolution is kept below 15 μm. In order to obtain either a 2D slice (B-scan) or a 3D (volume) tomogram, the beam is scanned transversally by galvanometric scanners *X-Y*. The signal is visualised in real time as a cross-sectional view and stored for postprocessing. The numerical processing of the data, besides the reverse fast Fourier transform, essential to the SOCT method, includes: subtraction of noninterference background, spectral shaping, and numerical dispersion correction.

**Figure 15.**
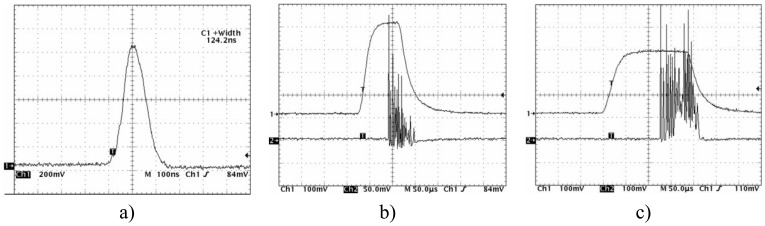
Time dependence of the Er:YAG laser pulses for different generation regimes: a) Q-switching – 125 ns with output energy 11 mJ; b) short free running - 60 μs burst of about ten pulses of 1 μs duration with output energy 10 mJ; c) free running – 120 μs burst of dozens pulses of 1 μs duration with output energy 21 mJ.

An essential prerequisite for laser cleaning is the proper choice of laser wavelength. This depends on the absorption coefficient of the material to be ablated. In the experiments described, an infrared Er:YAG laser (ReNOVALaserb 2940) working at a wavelength of 2.94 μm was used to ablate various varnish layers from fused silica plates. Radiation of this wavelength is strongly absorbed by the OH bond vibration (the absorption coefficient can reach a value of as high as 10^5^ cm*^-^*^1^), so that the depth of penetration of light into the treated medium may be extremely small [22]. Three pulse operation modes of laser were tested (Figure 15): Q- switched, short free running and free running generation.

**Figure 16.**
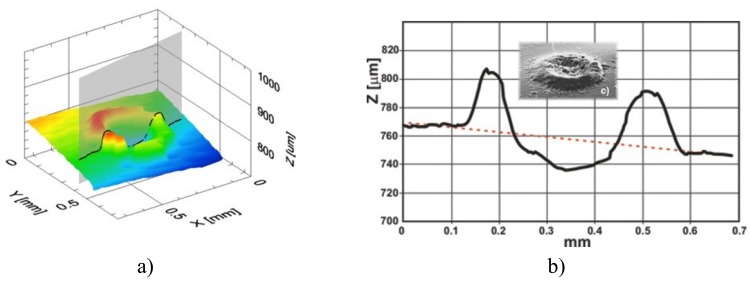
OCT analysis of Er:YAG ablation crater in varnish: a) recovery of shape; b) OCT measurement of the crater depth; c) SEM crater image – small photograph in b). In this case excessive laser energy density causes varnish outflow.

**Figure 17.**
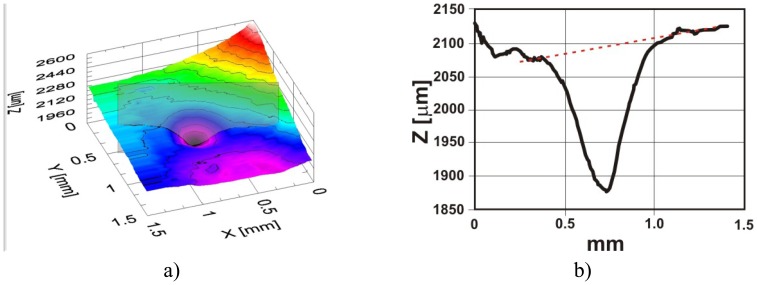
OCT analysis of Er:YAG ablation crater in varnish: a) recovery of shape; b) OCT measurement of the crater depth. Proper matching of laser fluence.

The OCT method enables two kinds of analysis of the ablation craters made with an Er:YAG:

- qualitative: the shape of craters (surface map) is recovered, additionally (SEM) images were made to compare to the surface maps generated from volume OCT data (Figure 16a,c, 17a),
- quantitative: the depths of the ablation craters are measured using OCT data (Figure 16b, 17b).

**Figure 18.**
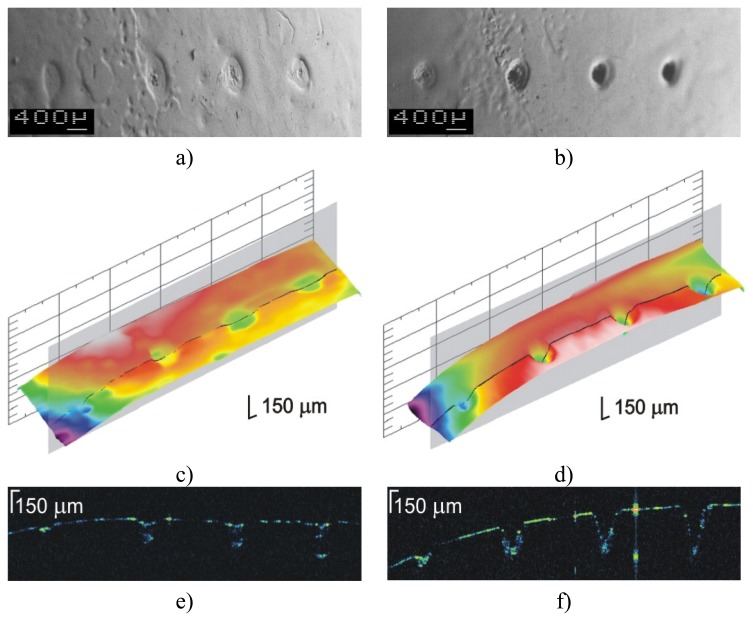
Testing of ablation conditions of Polyester resin in styrene without matting agent with two irradiation fluences: a,c,e) 4 J/cm^2^, and b,d,f) 7 J/cm^2^. In both cases, craters were formed by accumulation of 1, 2, 5, and 10 laser pulses (from the left to the right). a,b) SEM surface microphotographs; c,d) surface maps recovered from OCT data**; e,f**) OCT tomograms (B-scans).

**Figure 19.**
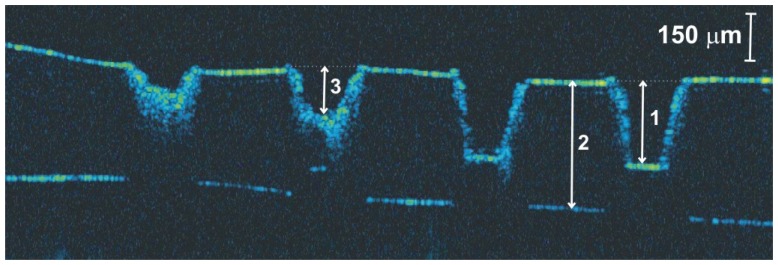
The OCT tomogram of the ablation of polyester resin in styrene with SiO_2_ as a matting agent using a fluence of 7 J/cm^2^. Craters were formed by accumulation of 1, 2, 5, and 10 laser pulses (from the left to the right).

To prepare varnish for quantitative OCT examination, a set of graded craters was produced by directing cumulative numbers of impulses of the same fluence to consecutive locations in the sample. In these experiments, two sets of craters were created for each sample: one with a fluence of 4 J/cm^2^ (Figure 18(a)), the other with a fluence of 7 J/cm^2^ [Figure 18(b)]. As can be seen from Figure 18, there is excellent correspondence between the microphotographs and the surface profiles obtained with OCT. However, extra useful information may be obtained from the OCT tomograms. Firstly, the surface profile is clearly visible, allowing tracking of the ablation process: the depth of crater was chosen as an indicator of progress. Secondly, the range of alteration of the structure of material under the crater surface is seen—this feature is not accessible with SEM. Thirdly, it is clearly visible when the whole layer is burnt through (Figure 19).

As it can be seen in Figure 19, craters formed by 5 and 10 pulses reach the substrate surface, and determination of putative crater depth is not possible. The discrepancy between the thickness of the resin layer in the craters (1) and in the resin itself (2) arises because of refraction in the resin, and vertical distances recovered by OCT are optical ones. The bars indicate distances in the air – proper crater depth (3).

#### 3.2.3. Laser interferometry

**Figure 21.**
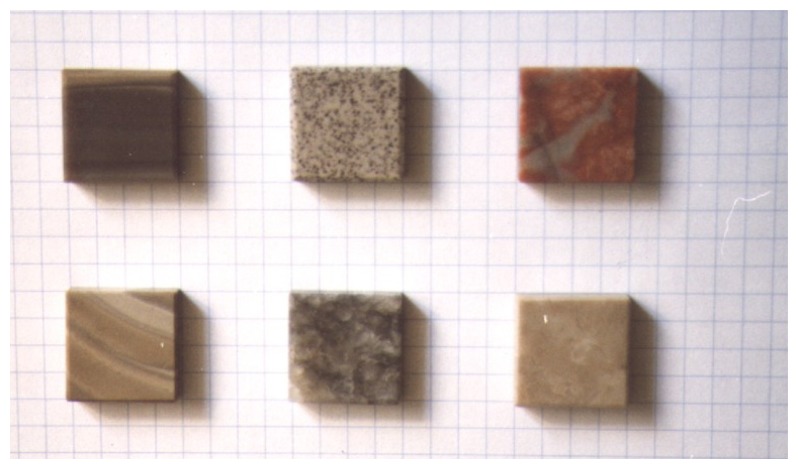
Photographs of different stone samples with dimensions of 4×20×20 mm^3^.

**Figure 22.**
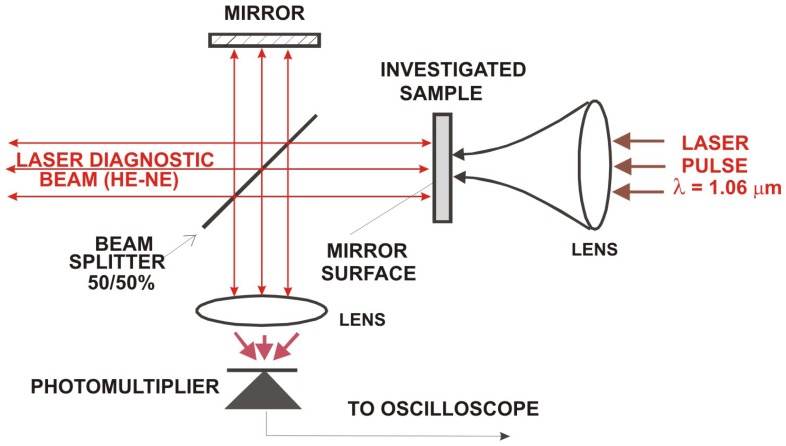
Scheme of experimental arrangement with Michelson interferometer.

**Figure 23.**
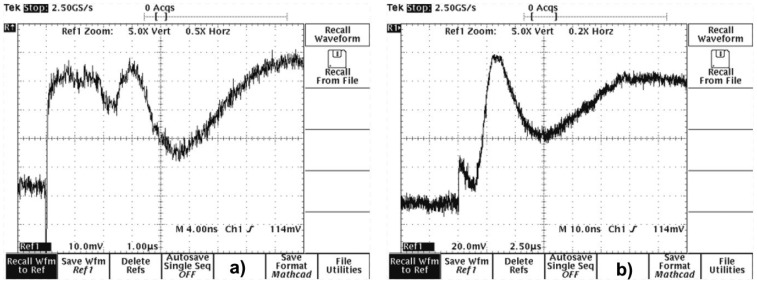
Oscillograms of shock-wave generated in stone samples by intense pulse laser irradiation: a) fluence 2.5 J/cm^2^; b) fluence 4.5 J/cm^2^.

Laser interferometry is a well-established, highly sensitive technique for non-destructive testing and analysis. It can be useful in the field of historical object conservation in reconstruction of 3D structure of the object under observation and its deformation under stress [23]. A typical example of application of laser interferometry is the study of externally induced vibrations of frescoes for individuation of defects, layer-to-layer detachments, delaminations and surface cracks [24]. On a smaller scale, interferometric techniques can be used for precise determination of surface profiles and study of small changes of the object in time. As it is shown in Figures 21, 22 and 23, sensitive laser interferometry can be also utilized for “on-line” analyses of shock waves generated during laser cleaning of quite thick objects [25]. As the amplitudes of registered shock wave pulse decrease with the decay of soiling layer, it can serve as an indicator of laser cleaning level. At the other side, substantial signal increase may warn against possible hidden defect in the structure of fragile object and stop the laser. This technique may be useful particularly during the “on-line” diagnostics of cleaning of thin and fragile objects.

In the experiment, six samples of stone materials (granite, marble and sandstone) were specially prepared for studying of generation of shock waves during interaction of high intensity pulse laser radiation (Figure 21). The experimental arrangement is shown in Figure 22.

One, front surface of stone samples was polished and served as a reflection mirror in typical Michelson interferometer, with He-Ne laser as a source of coherent radiation. The back, mat side of each sample was irradiated using focused beam of Q-switched, Nd:YAG ReNOVALaser 2 system (1.06 μm) with output energy of 100 mJ and pulse duration of 8 nsec. The value of fluency was controlled by means of distance between focusing lens and back side of sample. The vibrations of sample surface under the interaction of intense laser beam caused time variations of the intensity of interference fringes. The intensity changes were collected by photomultiplier and registered by TDS620 oscilloscope. Two chosen oscillograms for different laser fluences are shown in Figure 23. In all cases, calculated maximal movement velocity of samples free surface was lower than 30 cm/s.

### 3.3. Optical and physical methods

#### 3.3.1. Thermography

**Figure 24.**
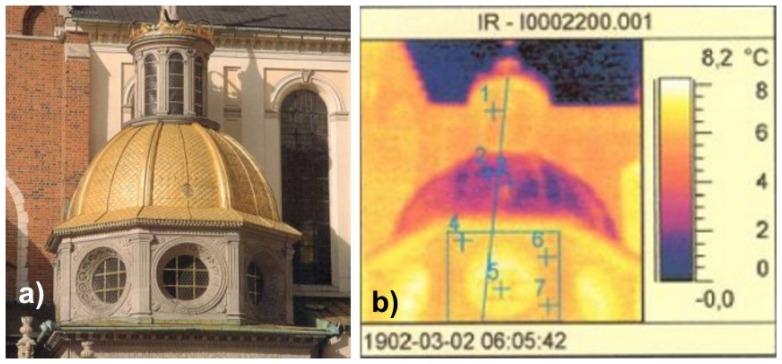
a) View of upper part of the Sigismund Chapel; b) Temperature measurement of lantern, dome and tambour. Photo by J. Marczak.

**Figure 25.**
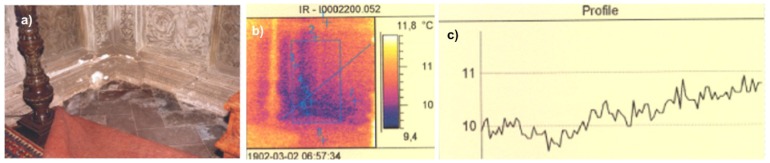
a) Preservation condition of southern-eastern bottom corner of the Sigismund Chapel; b), c) Results of thermographic measurements: b) thermogram; c) temperature profile along the diagonal line from b).

Thermographic investigations are applied everywhere, when determination of temperature distribution or object emissivity could be useful for evaluation or forecasting of a phenomenon or for detection of interesting for us object characteristics. Infrared thermography is considered to be of the greatest importance in the investigation of historic structures, where a restoration or conservation treatment can cause irreversible damage to the structure [26]. Moreover, thermovision technique is used for evaluation of thermal isolation of historical buildings, for detection of hidden defects under plaster or mosaic layer, delamination mapping, in testing of overheated electric installation as well as during laser removal of encrustation from art works and for monitoring of substrate temperature [27,28]. Figures 24, 25 show example results of thermographic visualizations of parts of the Sigismund Chapel at Wawel Cathedral, Cracow.

**Figure 26.**
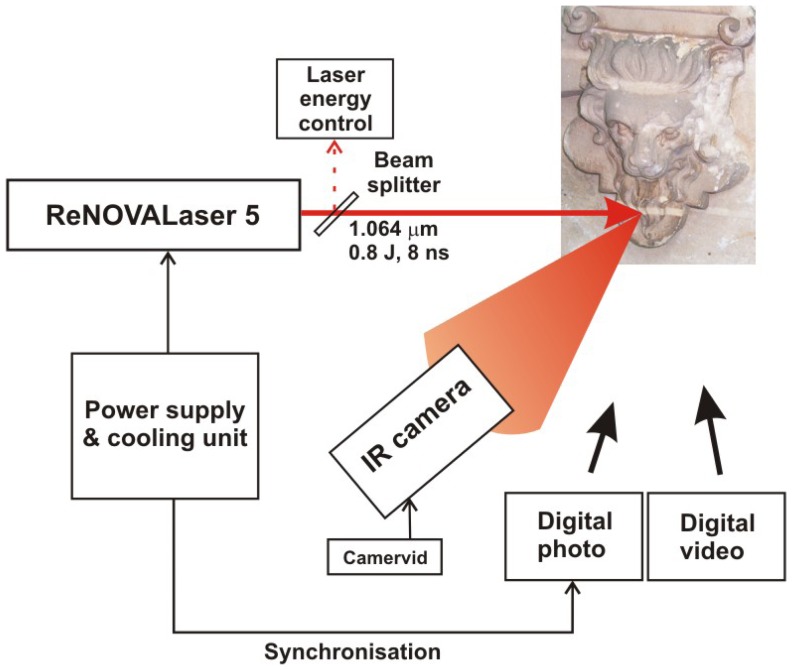
Scheme of experimental setup during thermographic studies of laser cleaning.

The main task of studies presented below was evaluation of the dynamics of thermal effects on the surfaces of various materials exposed to different laser stimulations. The aim of this preliminary work was to check infrared thermography as a supporting tool for many others techniques applied for optimisation of cleaning procedures using very short laser pulses. Experiments were performed with AGEMA 900 SW and LW infrared cameras. Images were acquired and digitally stored at the 15 Hz acquisition frequency (66.7 ms sampling time). Usually, there are only separate regions of interest, the same among hundreds of thermograms. Transformation of thermograms to the single synthesized image (“*the field dynamic thermogram*”*)* resulted from a few steps of data processing, starting with studies of whole sequence to determine the most representative sectors and limits of further presented fields (ARO). Selected parts as a set of data were exported next to the spreadsheet. Both procedures were realized utilizing camera integral software ERICA and MatLab™ software. Averaging of consecutive rows of ARO to the final form of one thermo-profile line of the same length in number of pixels as the ARO field, allowed a reduction of noises and other adverse effects [28]. Typical experimental setup is shown in Figure 26. Experiments were carried out with the use of Nd:YAG, Q-switched ReNOVALaser 5 system (800 mJ, 8 ns, 10 Hz, generating wavelengths of 1,064, 523, 355, 266 and 214 nm).

**Figure 27.**
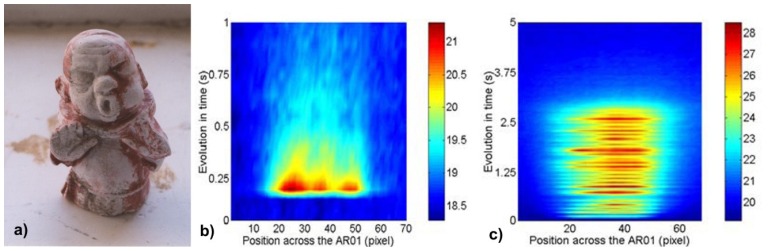
a) Cleaned Buddha statue (painted gypsum); b) Field dynamic thermogram (FDT) for one laser pulse (centre); c) FDT for a 3s train of laser pulses.

Two kinds of laser ablation regimes were realized during cleaning tests of a gypsum Buddha statue (Figure 27a). The first presented thermal effects of the one pulse of 600 mJ energy and 10 ns duration (Figure 27b), and the second shows thermal effects induced by the pulse train of about 3 s long (Figure 27c). In both examples the heating effects of ablation can be clearly seen. Differences in temperature distributions presented in Figure 27b result mainly from differences of absorption spatial features. In the case of cyclic irradiation reported in Figure 27c, only local heating effect is noted (low lateral heat diffusion). Frequency of pulses was low enough to protect surface against dangerous high or long-term overheating.

**Figure 28.**
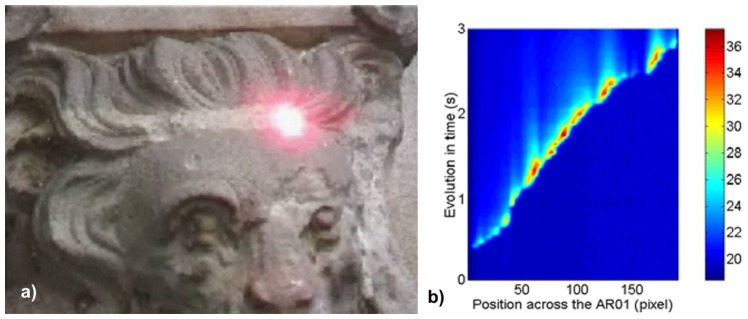
a) Frame from laser cleaning movie showing ablation spot at the surface of sandstone sculpture of lion; b) Field dynamic thermogram image.

Presented in Figure 28a, the cleaning of a sandstone sculpture of a lion was conducted to show an application of dynamic infrared thermography to study temperature evolution induced by scanning of the pulsed laser beam along the sandstone target. This time, constant was the speed of laser spot movement, i.e. about 10 cm/s in 3 s train. In practice, the speed of cleaning and the energy of irradiation are locally matched to safety thresholds and ablation results i.e. monitored by the operator supported with spectroscopic or acoustic instruments. The photograph in Figure 28a represents one frame from digital movie synchronized with laser pulse (red spot). Figure 28b shows FDT during movement of laser spot, resulting from MathLab™ software.

**Figure 29.**
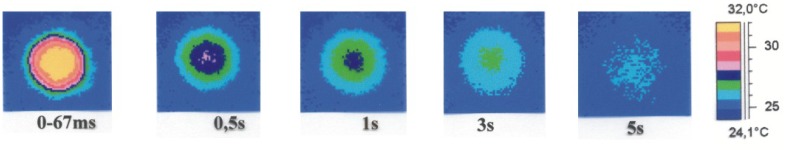
Rate of substrate temperature equalization (sandstone) after illumination by a laser pulse.

Temperature evolution after single pulse ablation for the same sandstone substrate is shown in Figure 29.

**Figure 30.**
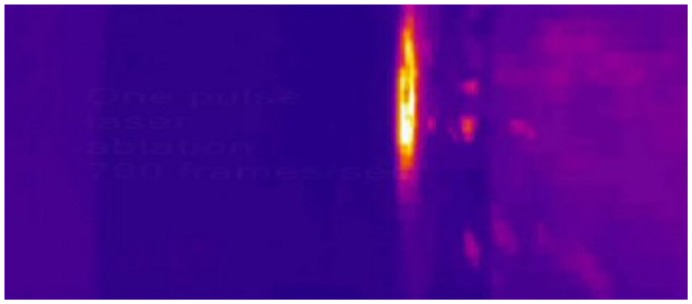
Ejection of excited, “hot” particles, registered during one pulse ablation of sandstone.

Figure 30 illustrates (as a curiosity) creation of plasma during laser ablation registered over object surface. It shows one frame selected from longer movie (700 frames/s) registered after one pulse interaction with sandstone.

#### 3.3.2. Analysis of light diffuse reflection coefficient

It is known that encrustation is non-homogeneous along the whole object surface, and doesn't typically possesses equal thickness, structure and even colour. One of few physical parameters allowing description of encrustation characteristics is the average reflection coefficient of backscattered white light (or laser light). Spectrometric measurement of amplitude of diffusively reflected white light in the function of wavelength represents synonymous and objective colorimetry, frequently used for fast and convenient determination of cleaning level of different substrates [29]. Similar analyses allow also to match laser radiation wavelength to the sufficient absorption coefficient of layers to be removed.

**Figure 31.**
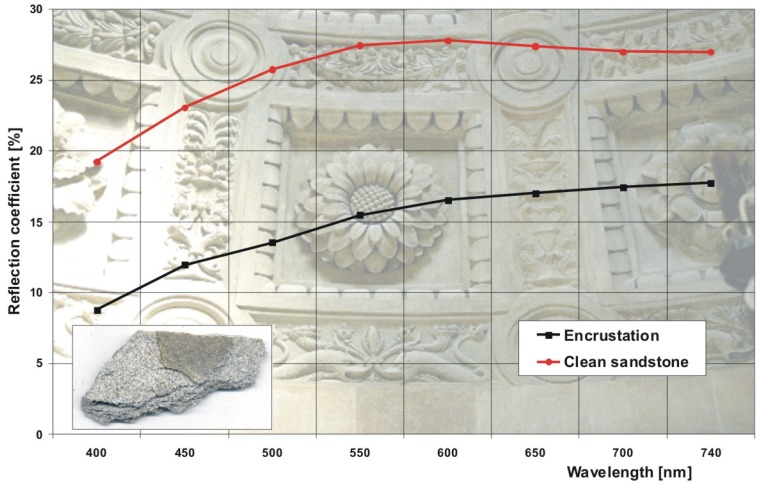
Reflection coefficient of two areas of Myślenicki sandstone in the function of light wavelength. Left bottom corner – sandstone sample photograph. Diagram background – sandstone flower ornaments in the Sigismund Chapel.

The small photograph in the left bottom corner in Figure 31 shows a sample of Myślenicki sandstone from Sigismund Chapel with clear separation between clean area (fracture) and surface with original encrustation. The complete chart presents the results of sandstone reflection measurements using a Konica Minolta CM2600d spectrophotometer.

**Figure 32.**
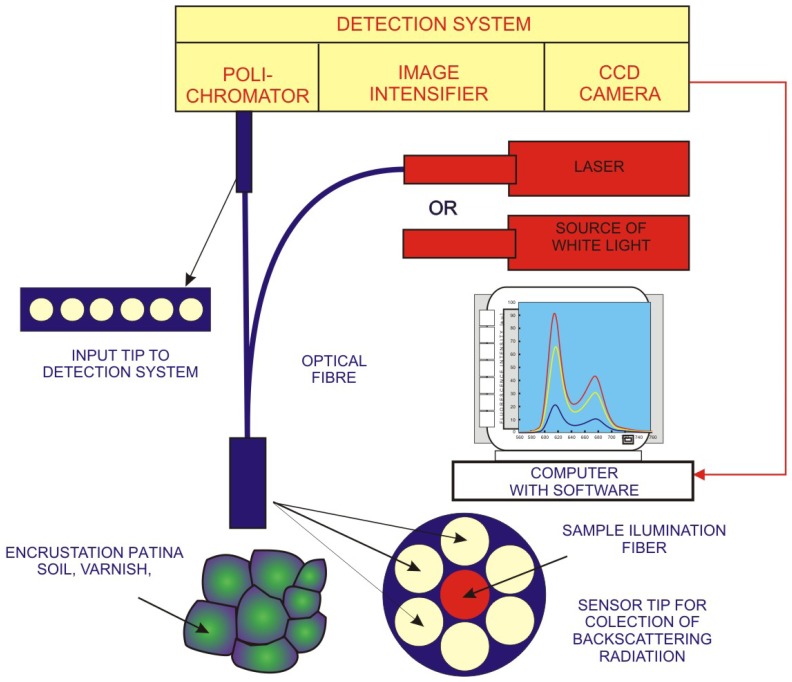
Scheme of diagnostic system with a fiber optics spectrograph for investigation of reflection (scattering) coefficient of superficial layers.

Figure 32 presents more sophisticated measurement system, developed for precise measurements of spectral reflectivity to determine laser cleaning levels. The task of fiber optics spectrometer (FORS) shown in Figure 6 was detection of backscattered light amplitude in the place of cleaning. Light emitted by halogen lamp (mercury, sodium lamp or even another laser) was delivered to the cleaned place of object by central optical fiber. Backscattered light is collected by six other fibers surrounding the central one. Collected light is then transmitted through optical system to diffraction grating of spectrometer and, after dispersion, to the linear matrix of CCD detectors. Finally, it is displayed on the monitor of computer. The distance of measurement tip from examined surface was selected in such a way, to obtain a maximum of scattered light from clean object area.

**Figure 33.**
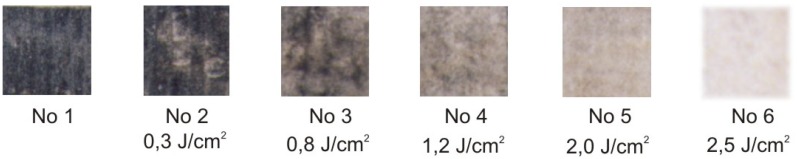
Illustration of successive stages of sandstone cleaning in dependence on irradiation level.

Figure 33 shows six squares (each with dimensions of 1.5×1.5 cm^2^), representing effects of sandstone surface cleaning with the use of Q-switched Nd:YAG laser, generating wavelength of λ= 1.064 μm. Square No 1 represents original colour of encrustation. In turn, squares from 2 to 6 represent cleaning results for increasing irradiation level, respectively: 0.3 J/cm^2^; 0.8 J/cm^2^; 1.2 J/cm^2^; 2 J/cm^2^ and 2.5 J/cm^2^.

Magnesia (MgO) is one of the best scattering materials and its scattering indicatrix almost ideally fulfil Lambert's law. In the presented case, MgO indicatrix determined reference for comparison of other values of light scattering in dependence on, for example level of cleaning of investigated surface. For irradiation level of about 2.5 J/cm^2^, colour of square No 6 is only slightly darker than the colour obtained from measurements of scattered white light in the place of fracture of sandstone (red line in Figure 24). Consultations with experienced restorers have confirmed that irradiation of 2.5 J/cm^2^ removes encrustation, together with the original sandstone patina.

**Figure 35.**
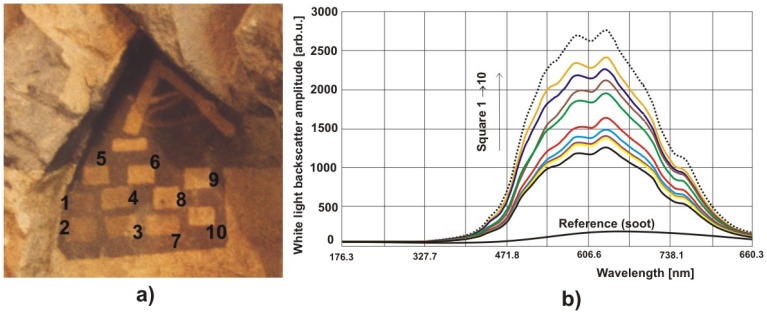
Results of measurements of light amplitude scattered from a fragment of limestone sculpture of Christ Pantokrator (Figure 3): a) photograph of measurement squares; b) experimental results.

**Figure 36.**
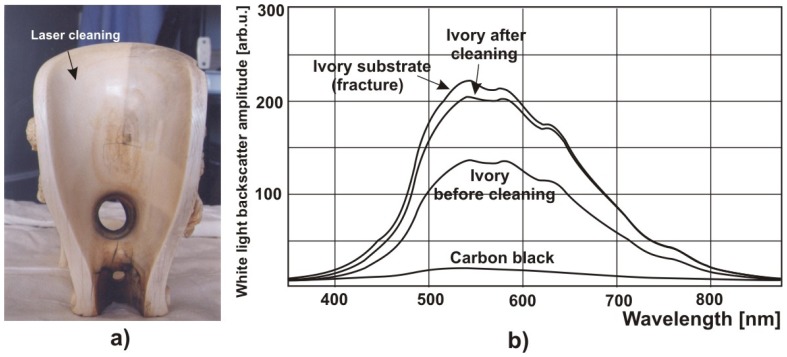
Results of measurements of light amplitude scattered from an ivory artwork: a) photograph of internal surface of ivory pitcher; b) experimental results.

Results of other similar measurements of different materials are shown in Figures 35 and 36.

#### 3.3.3. Color measurements (colorimetry)

Spectral reflectance data (Subsection 3.3.2) can also express color rankings by means of tone (hue), clarity (brightness) and saturation (chromaticity). Determination of their scale creates the possibility of digital (i.e. objective) and convenient color measurement.

Numerical color presentation methods were developed by international organization working with light and color – the Commission Internationale de I'Eclairage (CIE). Two well known methods are: color space YxY introduced in 1931 and color space L*a*b* introduced in 1976 and based on defined by CIE color modules XYZ [30]. Color space L*a*b*, determined also as CIELAB, is now one of the best known and wide used in almost all domains for object color measurements. Also the well known empirical test of laser cleaning efficiency relies on comparison of object color in dependence on laser fluence [31]. Additional attribute is documentary notation, which determines reference point and will allow to return to same hue after few dozen of years during next renovation procedures.

Table 3 contains colorimetric data obtained with the same as in the Subsection 3.2.3 Konica Minolta CM2600d spectrophotometer in the three experiments: laser cleaning tests of Gotlandic sandstone, and in two places of throne wall of the King's Batory Chapel in Wawel Cathedral, Cracow [9,32]. L* describes the brightness, a* the red-green color and b* the yellow-blue color. Right column of Table 3 shows photographs of investigated stone objects.

**Figure 37.**
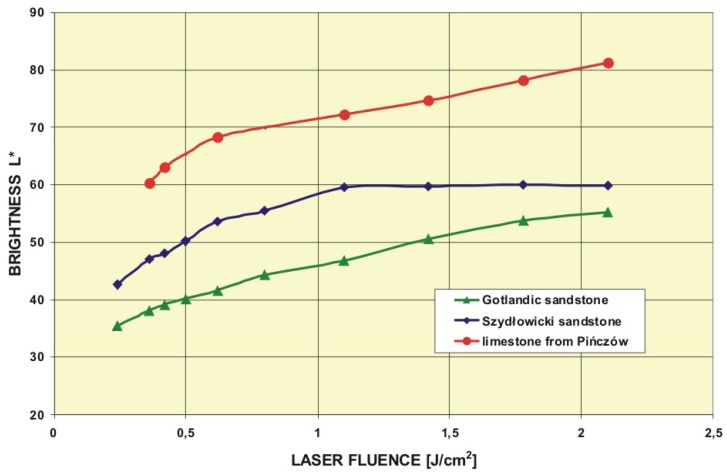
Dependence of the brightness L* on laser fluence in the laser cleaning tests of three objects presented in Table 3.

**Figure 38.**
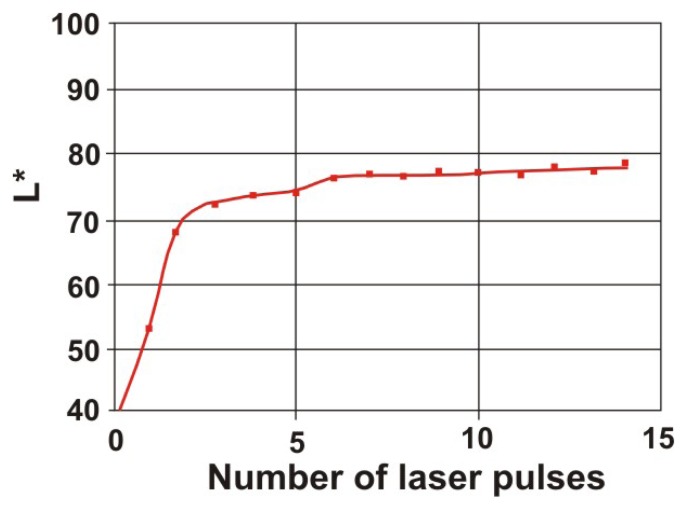
Dependence of the brightness L* on the number of laser shots (each 0.12 J/cm^2^) in the laser cleaning of gypsum.

Results presented in Table 3 are summarized in Figure 37. Value of the L* coordinate follows the increasing energy density of laser radiation applied to the consecutive square areas. In case of Szydłowicki sandstone, total encrustation removal has been obtained in the 7^th^ square area, for laser fluence slightly higher than 1 J/cm^2^. In the similar experiments with a gypsum sample (Figure 38), threshold laser fluence was around 0.7 J/cm^2^ (six laser pulses, 0.12 J/cm^2^ in each pulse).

**Figure 39.**
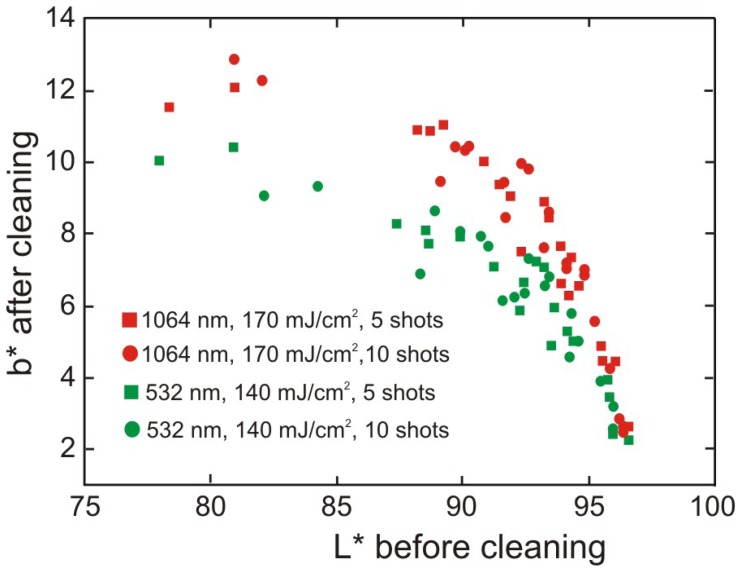
Yellowing of the substrate after laser cleaning as a function of density of the deposited soiling. Higher b* denotes more yellow component, lower L* denotes higher soiling density. Pulse repetition rate of 10 s^-1^.

It was already shown [33] that colorimetric results can be utilized in the assessment of laser cleaning of delicate, organic substrates like paper and parchment. The results of such experiment are shown in Figure 39. The discolouration of paper generally increases with increasing density of surface soiling (decreasing L*) while additional laser shots have no influence on the discolouration even at 532 nm. This wavelength seems to be preferable for pure cellulosic fibres and for removal of carbonaceous soiling and dust.

**Figure 40.**
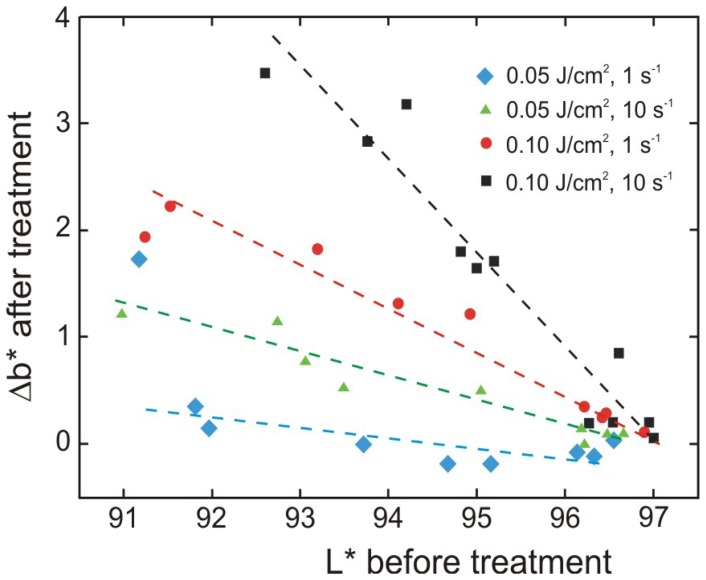
Yellowing of the substrate after laser cleaning at the conditions as indicated (Nd:YAG, 1064 nm), as a function of density of the deposited soiling. Higher Δb* denotes more yellow component, lower L* denotes higher soiling density.

Considering that cellulose is a thermal insulator and that heat is probably accumulated in the area close to the impact, it should be preferable if ample time is given for the material to cool down before the next laser shot at the same spot. It is confirmed in Figure 40, presenting similar results obtained for 50 laser shoots of 0.1 and 0.05 J/cm^2^, with frequency of 1 s^-1^ or 10 s^-1^.

Regardless of the data scatter, the results show that lowering the frequency decreases the intensity of discoloration, while still retaining the desirable cleaning effect. Especially experiments with the lowest fluence 0.05 J/cm^2^ lead to almost no yellowing detectable by the human eye (Δb* < 1) at low densities of surface soiling. However, it has to be stressed that these working parameters are certainly not universal and should be checked for the particular type of soiling and type of substrate in practical cases.

#### 3.3.4. Analysis of acoustic wave amplitude

**Figure 41.**
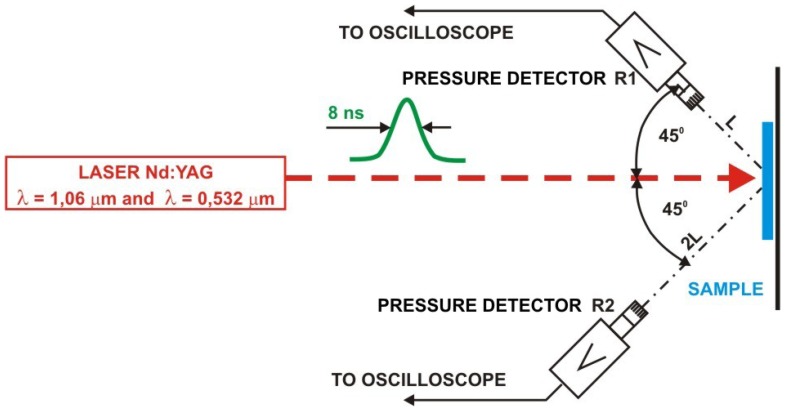
Experimental arrangement for acoustic assessment of the shockwave amplitude accompanying pulse laser cleaning.

**Figure 42.**
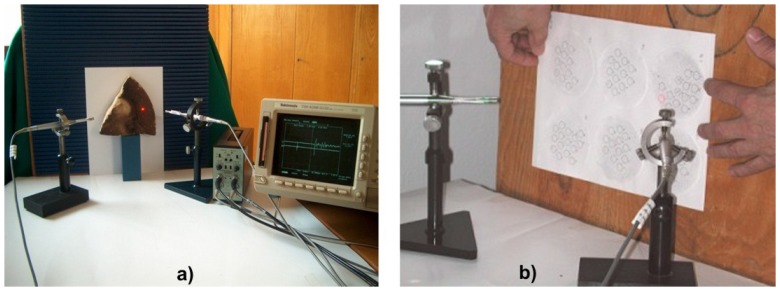
Photographs of experimental stand with microphones: a) cleaning of sandstone with natural ecrustation; b) cleaning of paper with artificial, normalized soil.

Laser cleaning, although with minimal side-effects, still needs to be monitored. Laser induced breakdown spectroscopy is among the most often used methods for this purpose, however, less costly alternatives would be welcome. Moreover, it is possible that LIBS evaluation of the cleaning effect during removal of soiling of unknown composition will be rather difficult [34]. Shockwave generation is a well-known side effect of plasma generation [35]. Using microphones with high dynamic range and monitoring the shockwave signals with an oscilloscope, the shockwave amplitude during each laser pulse may easily be monitored. Acoustic method of laser cleaning level evaluation has been applied in case of stone [36] and paper cleaning [33]. Experimental setup and photographs of experimental stand are shown in Figure 41 and Figure 42.

**Figure 43.**
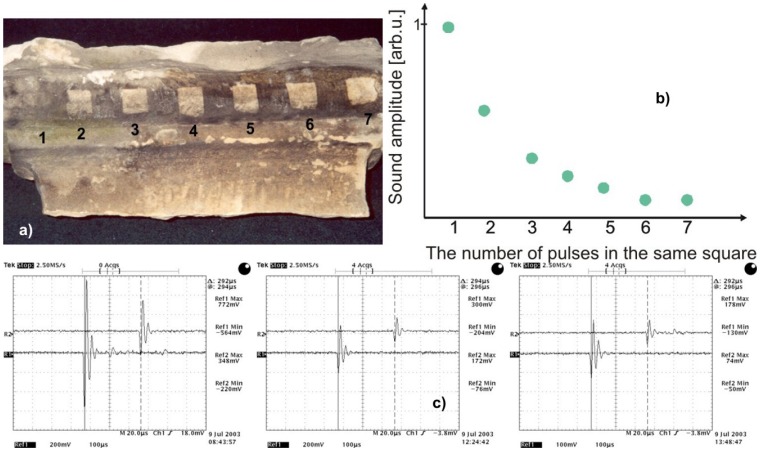
a) Photograph of a sandstone sample; b) Resulting dependence of acoustic signal amplitude versus number of pulses; c) Registered amplitudes of acoustic signals for first (left), fourth (middle) and seventh (right) laser pulse on the same square. Notice change of vertical scale for the last pulse.

Acoustic signal amplitudes presented in Figure 43c have been registered after first, fourth and seventh pulse directed to the same square area, as indicated in Figure 42a. It can clearly be seen that the shockwave amplitude decreases with an increasing number of laser shots on the same spot (Figure 43b). Since with each successive shot the substrate is cleaner, the cleaning process may be terminated at the desired level of cleanliness, thus matching the brightness of the laser-cleaned area to the predetermined earlier values, accepted by conservators.

**Figure 44.**
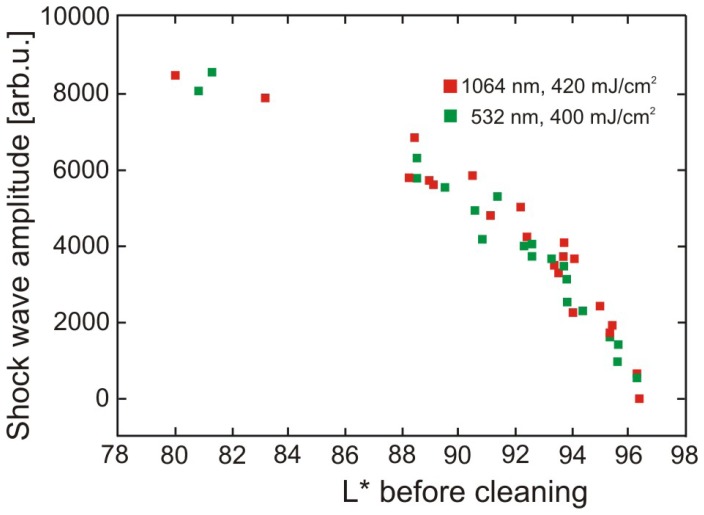
Shockwave amplitude after first laser shot at the conditions as indicated, as a function of density of the deposited soiling. Lower L* denotes higher soiling density.

**Figure 45.**
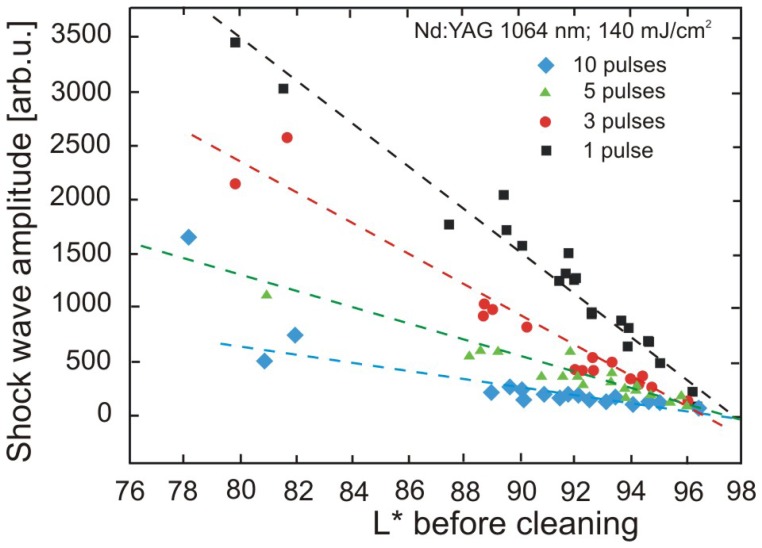
Shockwave amplitude after successive laser shots (as indicated) at the conditions as indicated, as a function of density of the deposited soiling. Lower L* denotes higher soiling density.

**Figure 46.**
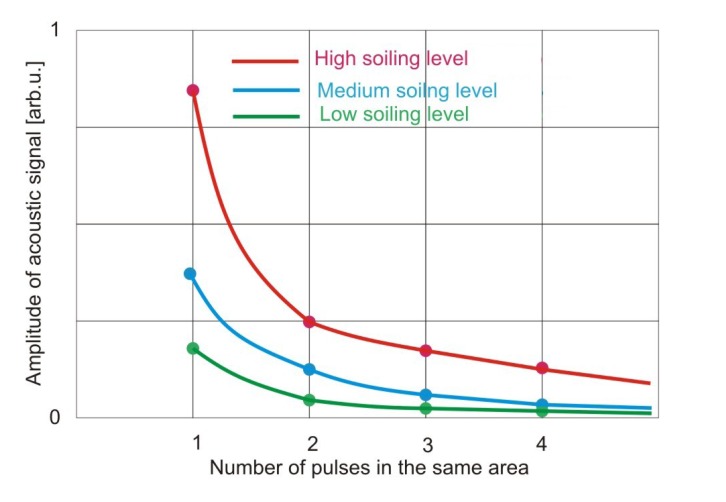
Amplitude of acoustic signal for three different soiling levels as indicated, as a function of the number of successive shots.

Results of paper cleaning experiments (Figure 42b) are shown in Figures 44, 45 and 46.

From the data presented in Figure 44 it can be concluded that the amplitude of the generated shockwave does not depend on the wavelength used. Considering that cleaning at 1,064 nm will lead to more surface discolouration, compared to 532 nm (Section 3.3.3, Figure 39), this methodology of on-line monitoring of laser cleaning cannot be used to estimate the inherent damage caused during the process.

However, in Figure 45 and 46 it can clearly be seen that the shockwave amplitude decreases with an increasing number of laser shots on the same spot. Furthermore, in the case of an intensive shockwave, removal of a dense layer of surface material is indicated, which might mean that the laser beam erroneously hit at an undesirable target, such as printing ink.

#### 3.3.5. Analyses of surface damage thresholds and roughness

The main aim of laser cleaning is removal of encrustation and reinstatement of primary appearance without violation of original material of artwork. In several situations described in the previous sections, analytical methods were always supported by “the eye” of experienced conservator. At the other side, conservation procedures frequently concern objects made of fragile and damage susceptible materials. In such cases, even if laser cleaning is sometimes an irreplaceable technique, application of **safe** laser cleaning techniques needs really careful determination of the substrate damage threshold – threshold laser fluence values. Moreover, even if the ablation process can be self-limiting, the energy density required for the removal of the coating should be lower than the predetermined threshold value above where damage of the substrate takes place. Furthermore, in many cases there is a lack of scientific reports describing values of safe doses of laser energy in the cleaning process.

A good example are bones and ivory, whose structure and physico-chemical properties generate problems for the determination of cleaning procedures [37]. The anisotropy and hygroscopicity of those materials exclude the utilization of aqueous solutions in their conservation. Acidic or basic solutions and organic solvents, as well as abrasive techniques, are also not recommended due to the possibility of artifact scratch or crumbling.

**Figure 47.**
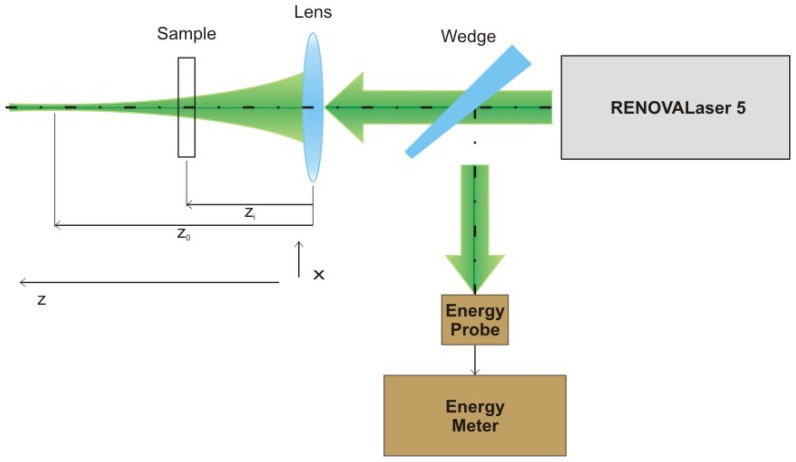
Experimental setup for determination of substrate ablation damage threshold.

The values of laser fluency which causes damage to the surface of bone or ivory sample, has been determined by means of *z*-scan method in the experimental set-up shown in Figure 47[38, 39].

A sample under investigation, placed in the path of laser beam close to the lens, was moved by 5 mm each time in the direction of beam waist plane (thus increasing energy density on the sample surface) and exposed to one laser pulse, until clear damage to the surface was seen with naked eye. The sample was then removed and examined with microscope to prove if the damage indeed occurred and to determine in which location. Then the sample was placed again in the laser beam path in the place shifted by 5 to 10 mm from position *z*_i_, in which damage was detected, and the sample was moved by 1 mm step by step in the direction of beam waist plane in order to locate the exact place, and therefore exact laser fluency, at which the damage occurs. It has to be mentioned that every sample was also moved crosswise to the axis of laser beam in order to achieve averaging on the surface, thus minimizing influence of potential heterogeneity or surface cracks on determined damage thresholds.

In the process of damage threshold determination for bone samples, the laser was operated mainly in a single-shot mode. However, when it was discovered that damage occurred in a certain position *z*_i_ corresponding with specific fluency, the sample was placed in the location shifted by 10 to 15 mm in the direction of lens thus decreasing energy density significantly, and was exposed to pulse laser radiation with repetition rate of 10 Hz. Afterwards the sample was moved in the direction of beam waist until sample surface damage occurred. Such procedures allowed for determination of whether there are differences between the effects of single pulses and a series of pulses with relatively high repetition rate, which are used in practice during laser cleaning of real objects. The RENOVALaser 5 system allowed for control of pulse repetition rate in the range from single shots to 10 Hz. The laser pulse width was constant and equal to about 15 ns (FWHM). Results of experiments are shown in Table 4, together with miniature black-white photographs of investigated samples.

**Figure 48.**
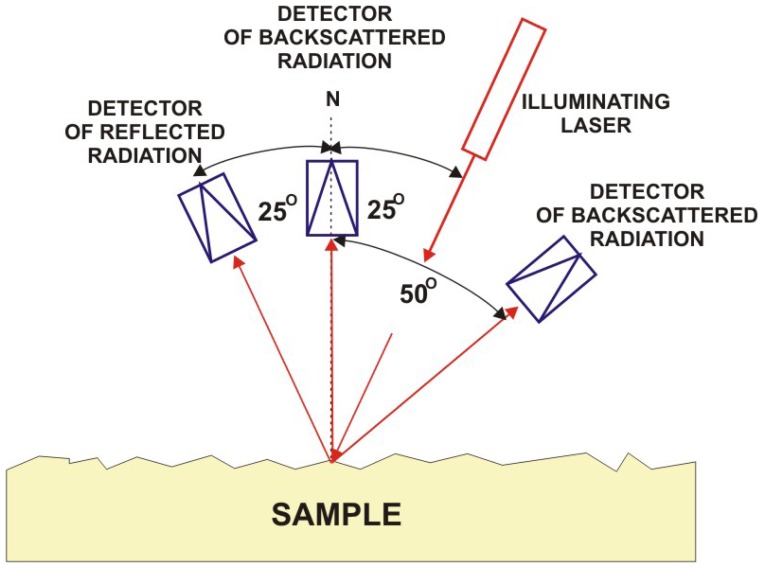
Experimental setup for the determination of artwork surface roughness.

The morphological changes of the surface of the samples can be assessed by means of a surface roughness measurement (Figure 48) [31, 36].

**Figure 49.**
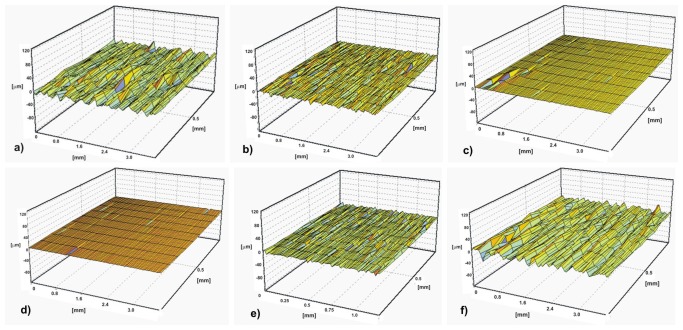
Surface roughness as a result of different cleaning techniques: a) glass beads; b) micro sandblasting; c) walnut shells; d) water blast; e) laser cleaning; f) corundum powder.

Comparison of surface roughness after different cleaning processes in the case of bronze object are shown in Figure 49. Laser scatterometer mScan SMS assures measurement range 3 – 1,177 Å with accuracy of 0.1 Å (semiconductor laser λ=670 nm).

The best surface quality, defined as the lowest roughness is attainable with the use of water blasting and abrasive cleaning with grinded walnut shells. However, these methods are not so effective in cleaning (especially in case of irregular surfaces) as the laser cleaning. Results, presented in Figure 49 are consistent with the results published in [40].

Roughness measurements are also standard studies of material's damage thresholds. The establishment of damage thresholds is based on variations of RMS roughness induced on surfaces after any kind of treatment, above the predetermined value, which represents the standard deviation of the RMS roughness values measured in polished, reference sample surfaces before treatment. Variations above these values are attributed statistically to the impact of cleaning treatments on surfaces. The word ‘damage’ is used rather freely, as the threshold values are based on the equipment resolution and on the magnification chosen for this set of experiments. Moreover, the correlation between surface modifications and surface damage is dependent upon many factors, connected usually with characteristic features of substrate materials and encrustation. Measured values represent the threshold above which variations in surface topography are statistically attributed to cleaning treatments. The correlation of these values with damage thresholds will depend upon the definition of damage imposed, and this will vary according to principles such as the perception of the conservator of the object. The general aim of such measurements is to monitor variations on surfaces and establish such threshold values, to define the levels at which relevant parameters are likely to increase surface roughness. These are meant to provide a general guideline to the use of cleaning treatments, whereby conservators will be able to predict the extent of surface modification during treatments. The aesthetic impact of studied cleaning procedures is usually verified by means of optical microscopy and quantified by colour measurement.

**Figure 50.**
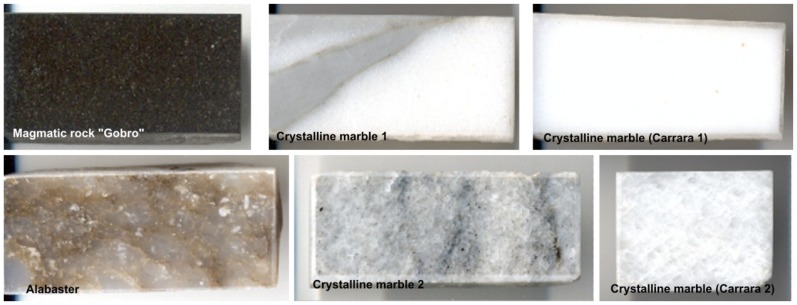
Photographs of stone samples before determination of laser ablation damage threshold.

Figure 50 shows six polished stone samples, for which laser ablation damage threshold has been determined. Measured average values of samples roughness and reflection coefficient are presented in Table 5.

**Figure 51.**
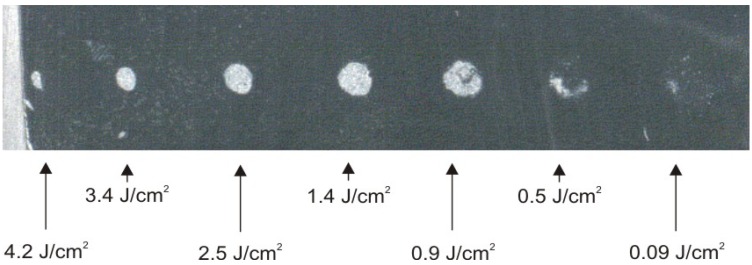
Photographic illustration of influence of different laser fluence on the surface of “Gobro” magmatic rock (Figure 50).

Measured laser threshold values for all samples fitted the range 80 – 200 mJ/cm^2^, with the lowest value for “Gobro” magmatic rock (80 mJ/cm^2^). Visual illustration of laser interaction with “Gobro” magmatic rock is shown in Figure 51.

## 4. Exploitation of the results, conclusions

Preservation of cultural heritage delivers to people priceless source of information about their history and civilisation and is one of the most important tasks for generations. Conservation of priceless historical objects is a set of complex and difficult problems. The main reason of increasing interest in applications of lasers and optoelectronics lies in plurality of advantages offered by these modern techniques, especially when such application of exact science can be critical towards art in order to help in the better understanding and cognition of cultural heritage.

The paper presents our modest attempt to describe and discuss analytical techniques applied to the diagnostics of art work, and particularly to characterize laser cleaning - comfortable and effective technique for the process of encrustation removal.

Laser Induced Breakdown Spectroscopy (LIBS) is one of the most promising and developed techniques for real-time control of the laser cleaning, in which the emission spectra of the locally generated plasma give important indications about the surface composition at the interaction zone [41]. Portable LIBS set-ups, suitable for field use, have been already developed and might easily be combined with a laser cleaning system based on the same Nd:YAG source. The LIBS technique allows for high spatial resolution (down to 10 μm) when performing single shot stratigraphy which is minimally invasive, requiring neither sample removal from its location nor its significant consumption. Despite these advantages the applications for LIBS are limited. Information about the sample is lost in the ablation process and only the elemental composition can be determined. Nothing is known about the molecular structure. The other main limitation of LIBS has been its qualitative or semi-quantitative nature, due to the so-called “matrix effect” (i.e. the strong dependence of LIBS spectra on relatively small variations of the material composition). This situation changed after the introduction of calibration free LIBS (CF-LIBS) spectra analysis [42].

A technique that gives information about molecular structure is Raman spectroscopy, which can be used to identify both organic and inorganic materials with comparable ease [43]. Raman microscopy is the ideal technique for the investigation of materials used on works of art because it is reliable, sensitive, specific, nondestructive and can be applied *in situ*, therefore avoiding any sampling and consequently any damage to the object under examination. A disadvantage of the technique is the possible presence of broadband fluorescence which may overwhelm the Raman signals, especially from varnished works of art such as oil paintings or icons. The use of optical fibers to guide both the laser light (red or green) and scattered light minimizes the power losses in comparison with the conventional Raman equipment [44]. Moreover, the mobility of the fiber probe (or optical head) allows to analyze almost any surface independent of its size, shape and location. Fourier-transform sensing in Raman spectroscopy offers advantage of the use of infrared excitation [45]. In this case the problem of the fluorescence of materials under analysis can frequently be overcome.

The majority of applications of Optical Coherence Tomography (OCT) concern the evaluation of paintings, particularly the topmost varnish layer. As it is shown in the Subsection 3.2.2, OCT permitted nondestructive, in situ and on-line estimation of varnish ablation speed and/or instantaneous monitoring of the thickness of remaining material. In principle however, the OCT idea allows noncontact and nondestructive imaging of any semitransparent objects. One of the first applications of OCT to investigate the structure of cultural heritage artefacts was the imaging of glaze layers, on a porcelain cup and on a faience plate [46]. It has been used also to monitor the subsurface morphologies of archaic jades [47] and to study the degradation of parchment caused by iron gall ink [48]. Recently, the new project has been launched to refine the OCT technique and extend the range of painted objects to include works on paper or textile substrates and wall paintings [49].

Stress measurements performed by the laser interferometry technique (Subsection 3.2.3) demonstrate that for all the analyzed samples ablation generates mechanical excitation of the structure. The high pressure induced in the laser irradiation spot propagates all over the samples bulk as vibrational modes and stress waves and can be qualitatively recorded even for relatively thick stone samples. This extended mechanical influence can provoke the appearance of delocalized defects formation after repeated impulsive irradiation, especially in the case of restoration of fragile materials. Laser interferometer can be easily replaced with vibrometric sensor [50].

The thermographic experiments presented in Subsection 3.3.1 revealed that the stone surface temperature increases by few degrees after a single laser shot. The temperature immediately (<67 ms) after firing of the laser can increase by about 15°C for a short period of time. This peak temperature is clearly related to the formation of the plasma plume and the temperature increase is directly related to the laser fluence. Presented method of direct determination of substrate temperature gradients could be more important in the case of temperature sensitive materials [51]. Beside presented effectiveness of infrared thermography as a non-destructive technique in the investigation of thermal isolation of historic structures, it can be also applied to evaluate conservation interventions [52].

Measurements of diffusively reflected laser or optical radiation included in Subsections 3.3.2 and 3.3.3 present only a small fraction of possibilities offered by studies of artwork reflectance. Its utility parameters are determined by the wide selection of sensors, sensing techniques as well as advanced software used. Spectral reflectance is utilized for characterization of cleaned areas [53], differentiation of white pigments [54], determination of maximum difference of absorption between the original material and encrustation [55]. Flexibility of measurements is supported by the use of optical fibers (Figure 32, also [54]) and substantial increase of spectral resolution by FTIR regime of spectra detection in the microreflectance mode [56].

Colorimetric representation of reflectance data in the CIE-L*a*b* color space allows the quantification of small differences in color (ΔE∗) below the minimum level of perception. It is utilized to study several conservation problems, including physical–chemical modifications of the surface induced by cleaning (laser irradiation), effectiveness of cleaning procedure, artwork ageing process as well as serves as a documentation of artwork surface digital color representation for future restoration works.

The morphological surface changes induced by laser irradiation or other cleaning method are frequently assessed by determination of roughness (Subsection 3.3.5). The roughness of the surface is evaluated mainly through measurements of RMS, the arithmetic mean deviation of the roughness profile but also through the maximum roughness depth [57] or mean value of roughness depth in several consecutive sampling lengths [58]. Although there are no widely accepted ways of correlating roughness and surface damage, methodology shown in [40] seems to be rational and has been adopted and described in Subsection 3.3.5 in application to the determination of stone samples damage thresholds. However, it should be noted that reduction in groove amplitude can be either the result of the cleaning products filling the bottom of the surface grooves or the damage process removing the peaks of the grooves. At the other side, observed small increase of the roughness after laser cleaning is frequently the result of selectivity and locality of laser ablation. It can't be ascribed to the disarrangement of surface structure by excessive laser energy density. Usually scanning electron microscopy (SEM, SEM/EDS), optical microscopy and colorimetry are used as the supplementary tools to examine how topographical variations correlate to the removal of surface material and, therefore, how cleaning methods interact with material surfaces. It was correctly pointed out in [59] that most of the evaluation methods, especially all those that involve measures of physical properties of the surfaces (color, roughness, etc.), can have significance only when different methods of cleaning the same surface are compared.

What should be remembered during design of laser cleaning procedure and development of diagnostic methodology? Each utilized laser arrangement is characterized by significantly different output parameters such as wavelength and pulse duration. Each individual object in turn creates its own and unique, different conservation problems. Hence, there is no possibility to prepare instructions, specific action procedures for conservator, determining the value of laser fluence, pulse repetition rate etc. that should be used for different encrustation and different objects. It is hence to be stated in this place that: **“each object should be treated individually, even moreover, individual areas of object should not be treated equally”.** Parameters, desired for specific encrustation removal and expected cleaning level are established during preliminary investigations. It should be also remembered that attainable quality of laser surface cleaning depends on the knowledge, manual predispositions and experience of conservator. Each object needs development of individual laser technology, which is determined during several conservation treatments carried out at different objects.

Numerical analysis that were carried out earlier [60-65], concerned quality results of determination of pulse laser beam influence on the behaviour of evaporated material. Developed physical model and computer software reveal high universality and correctly show the quality trends observed in all experiments.

Our list of restored and diagnosed objects contains more than one hundred of different monuments and artworks in Poland and abroad. The most representative and prestigious are the following works:

- conservation and laser restoration of internal décor of Sigismund Chapel at Wawel Hill in Cracow, about 800 m^2^ of decorative sculptor's surfaces,
- conservation and laser cleaning of walls and decoration elements in Arch-Collegiate Church in Tum, near Łęczyca, including ancient Romanesque sculpture of Christ Pantokrator,
- cleaning of elements of the Tomb of the Unknown Soldier in Warsaw,
- restoration of epitaph and stall of King's Batory Chapel at Wawel Hill in Cracow,
- cleaning of St. Blaise sculpture in Dubrovnik, Croatia,
- restoration of tombstone of Juliusz Słowacki and tombs of national government members “Avenue des Polonaise” at Montmartre Cemetery in Paris,
- cleaning of marble sculpture of Henryk Lubomirski in Łańcut (Antonio Canova, 1794).

## Figures and Tables

**Figure 3.**
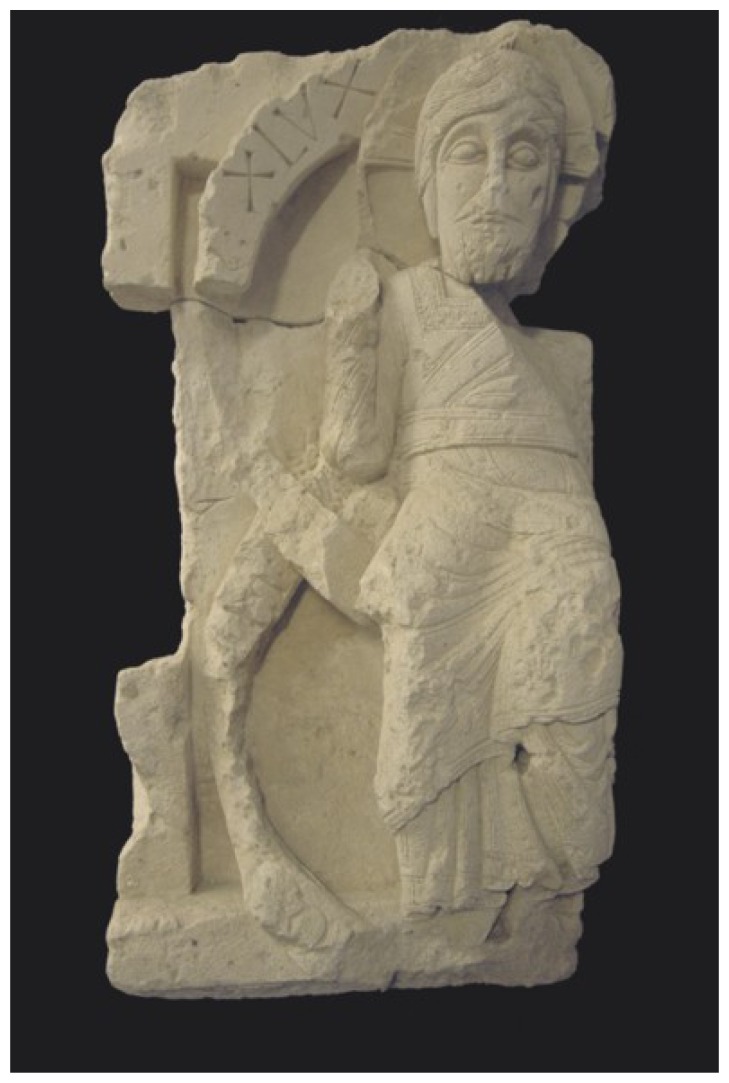
Christ Pantokrator (12^th^ century) after laser restoration. Arch-collegiate Church in Tum near Łęczyca, Poland. Photo by A. Koss.

**Figure 20.**
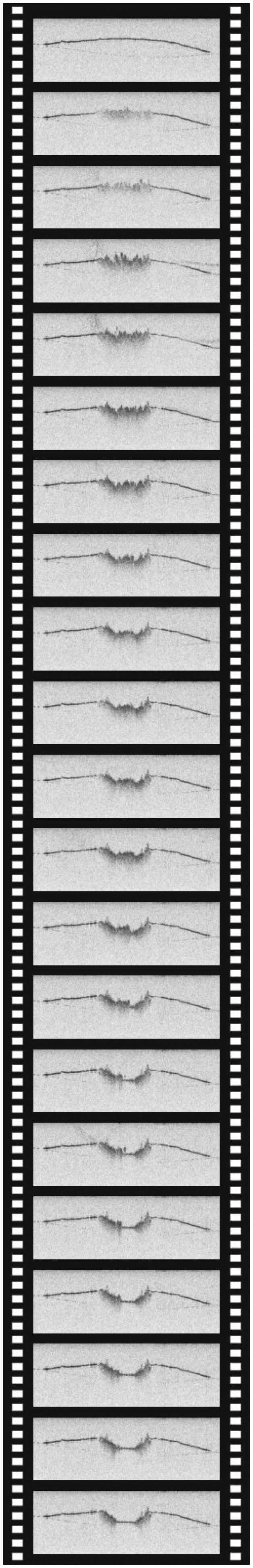
At the right side of page – frames, extracted from the OCT movie, registered immediately after interaction of consecutive Er: YAG laser pulses with varnish.

**Figure 34.**
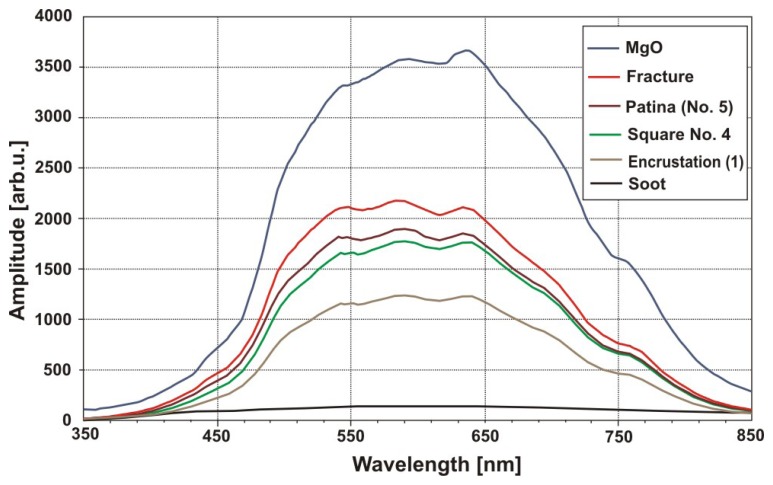
Results of measurements of light amplitude scattered from sandstone samples (Figure 33).

**Table 1.** Main atmospheric pollutants – their sources and influence on art works [13].

**Pollutant**	**Source**	**Influence**

Sulphur dioxide (SO_2_)	Combustion of fossil fuels, mainly coal	tarnishes metals degrades paints, dyes, photographic films, paper, parchment weakens fabrics
Nitrogen dioxide (NO_2_)	Motor exhaust gases, combustion in stoves, boilers, in industrial processes, decomposition of cellulose, photochemical reaction of other NO_x_	bleaches dyes weakens fabrics damages photographic films
Ozone (O_3_)	Photocopiers, laser printers, electrostatic particles filters, photochemical reactions	cracks rubber bleaches dyes degrades paper/parchment
Hydrogen sulfide (H_2_S)	Motor exhaust gases (catalytic converters), natural geochemical processes, vulcanized rubber, waterlogged organic materials	tarnishes metals, mainly silver
Carbonyls (formic acid, formaldehyde, acetic acid, C=O)	Drying paint, wood and wooden products, cellulose decomposition, resins, thermoplastics	causes corrosion of metal, particularly copper, zinc and lead alloys damages limestone, minerals and other materials
Solid particles	Traffic, abrasion, pollens, combustion, insects, salts	damages objects through deposition of acid/alkaline substances

**Table 2.** Lasers and optoelectronics in nondestructive analysis and diagnostics of monuments and artworks.

**DIAGNOSTIC METHOD**
**OBJECT STRUCTURE** **Chemical composition – surface analysis:** Reflection/Scattering Absorption/Transmission Laser induced fluorescence (LIF) Laser induced breakdown spectroscopy (LIBS) Raman spectroscopy **Physical structure (e.g. defects localization)** Multispectral imaging Interferometry, speckle interferometry Holography, holographic interferometry [17] Laser vibrometry [18] Thermography Laser tomography (OCT) **OBJECT MORPHOLOGY** 3D scanning – time of flight method Triangular scanning Surface profilometry Laser scatterometry Laser tomography (OCT)

**Table 3.** Color measurements of different stone substrates during laser cleaning tests.

**Gotlandic sandstone**									
Square No	Laser fluence [J/cm^2^]	L*	a*	b*	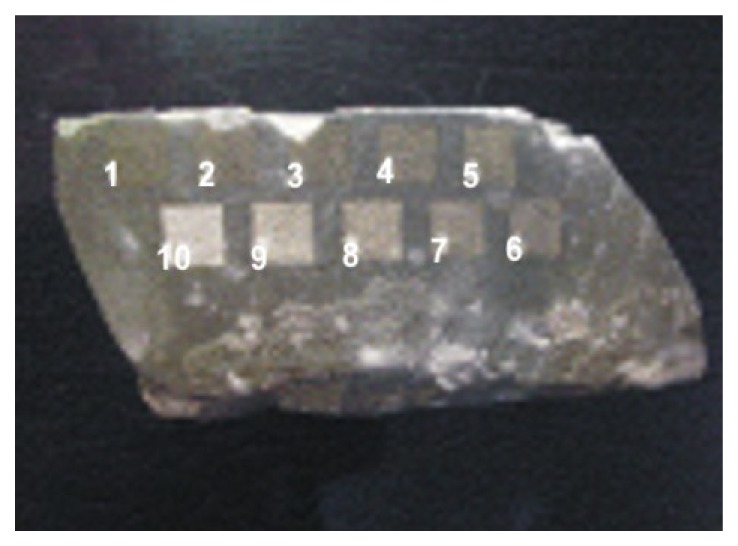
1	0.24	35.48	0.96	8.16	
2	0.36	38.12	0.92	8.98	
3	0.42	39.22	0.88	8.72	
4	0.50	42.11	1.02	9.03	
5	0.62	43.26	0.76	8.42	
6	0.80	44.32	2.14	14.12	
7	1.10	46.82	2.18	15.32	
8	1.42	50.64	2.26	15.94	
9	1.78	53.76	1.65	14.72	
10	2.10	58.39	1.25	15.76	
The King's Batory Chapel – throne wall, sculpture of angel (limestone from Pińczów, Poland)									
1	0.32	60.36	3.52	16	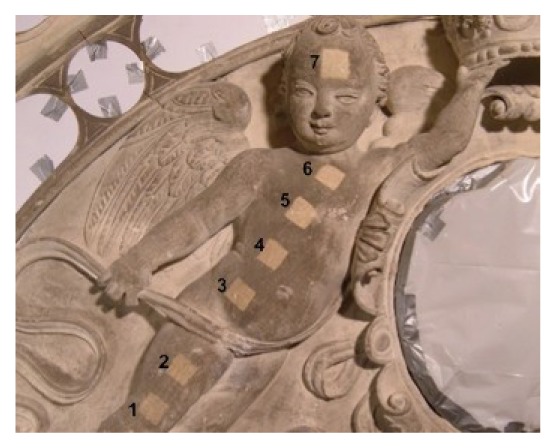
2	0.45	63.05	3.99	17.78	
3	0.68	68.31	3.69	17.63	
4	1.10	72.28	3.34	17.48	
5	1.43	74.66	3.28	17.81	
6	1.78	78.24	2.74	17.88	
7	2.15	81.22	2.12	15.40	
The King's Batory Chapel – throne wall, window frame (Szydłowicki sandstone, Poland)									
1	0.24	42.62	2.21	12.73	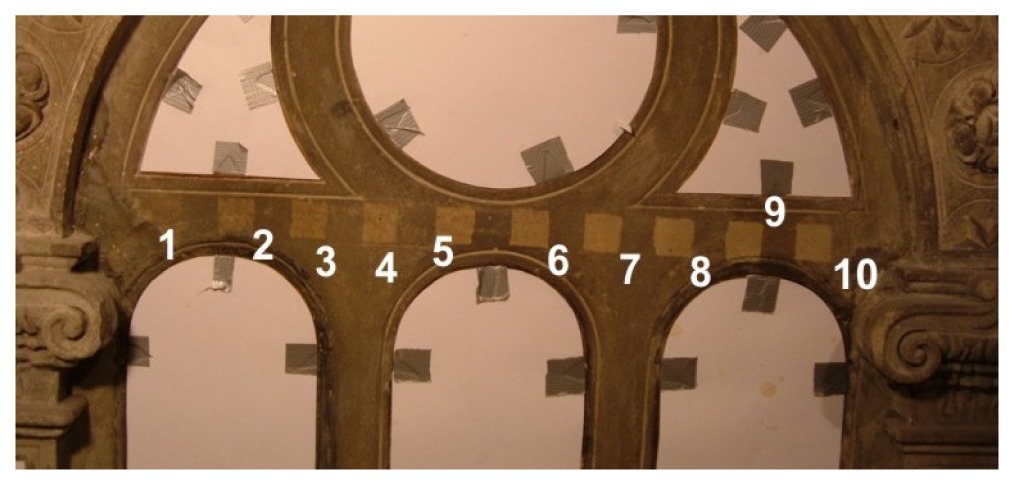
2	0.36	47.11	2.52	13.99	
3	0.42	48.06	2.96	16.20	
4	0.50	50.20	2.28	14.38	
5	0.62	53.66	3.01	15.81	
6	0.80	55.45	2.61	16.41	
7	1.10	59.56	2.89	19.08	
8	1.42	59.80	2.08	16.82	
9	1.78	60.02	2.08	16.53	
10	2.10	59.85	1.05	14.66	

**Table 4.** Damage thresholds of tested bone samples.

**Sample**	**Photograph**	**Damage threshold for single laser pulse [J/cm^2^]**			**Damage threshold for 10 Hz repetition frequency [J/cm^2^]**		
	
		**1,064 nm**	**532 nm**	**335 nm**	**1,064 nm**	**532 nm**	**335 nm**

“white” bovine rib	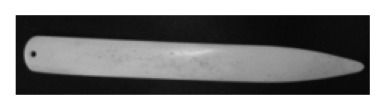	4.9	7.7	1	4.0	8.6	< 1.5
“brown” bovine rib	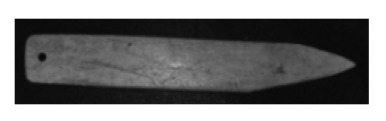	5.2	8.6	2.6	3.8	8.6	1
ivory (sample 1)	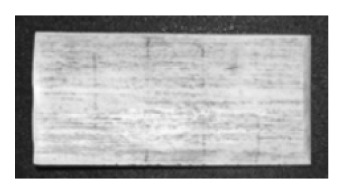	>20	18.6	3	7	5	0.9
ivory (sample 2)	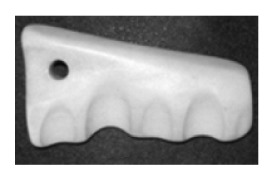	-	≫ 9	-	-	3.5	0.9
bovine horn	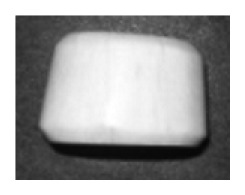	-	8	-	-	4.8	-
boar tusk	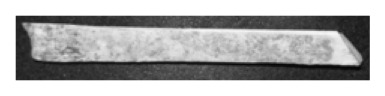	-	> 16	-	-	-	-
bovine tibia	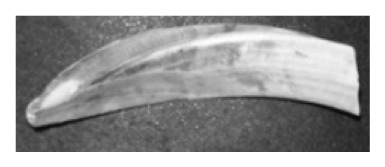	-	2	-	-	-	-

**Table 5.** Values of roughness (R) and reflection coefficient (RC) of samples in Figure 50.

**Magmaticrock “Gobro”**		**Crystalline marble 1**		**Carrara marble 1**		**Alabaster**		**Crystalline marble 2**		**Carrara marble 2**	
R[Å]	RC[%]	R[Å]	RC[%]	R[Å]	RC[%]	R[Å]	RC[%]	R[Å]	RC[%]	R[Å]	RC[%]
199.9	6.35	927.2	7.01	856.5	6.97	388.2	5.78	554.6	6.57	847.7	6.22
